# Supplementary material for: Rapid disintegration and weakening of ice shelves in North Greenland
Source: Nat Commun. 2023 Nov 7;14:6914. doi: 10.1038/s41467-023-42198-2 (PMC10630314; doi:10.1038/s41467-023-42198-2)
Supplement: Supplementary file 1 — Supplementary Information [file 41467_2023_42198_MOESM1_ESM.pdf]

Supplementary Figures for "Rapid Disintegration and Weakening of Ice Shelves in North Greenland"

R. Millan[1,*], E. Jager[1], J. Mouginot[1], M. H. Wood[2], S. H. Larsen[3], P. Mathiot[1], N. C. Jourdain[1], A. Bjørk[4]

[1] *Université Grenoble Alpes, CNRS, IRD, INP, 38400, Grenoble, Isère, France*
[2] *Moss Landing Marine Laboratories, San José State University, 95192, California, USA*
[3] *Department of Glaciology and Climate, Geological Survey of Denmark and Greenland (GEUS), Copenhagen, Denmark*
[4] *Department of Geosciences and Natural Resources Management, University of Copenhagen, 1350, Copenhagen, Denmark*
[*] *Corresponding author: romain.millan@univ-grenoble-alpes.fr*

File content.

This file contains supplementary Figures S1-S40.

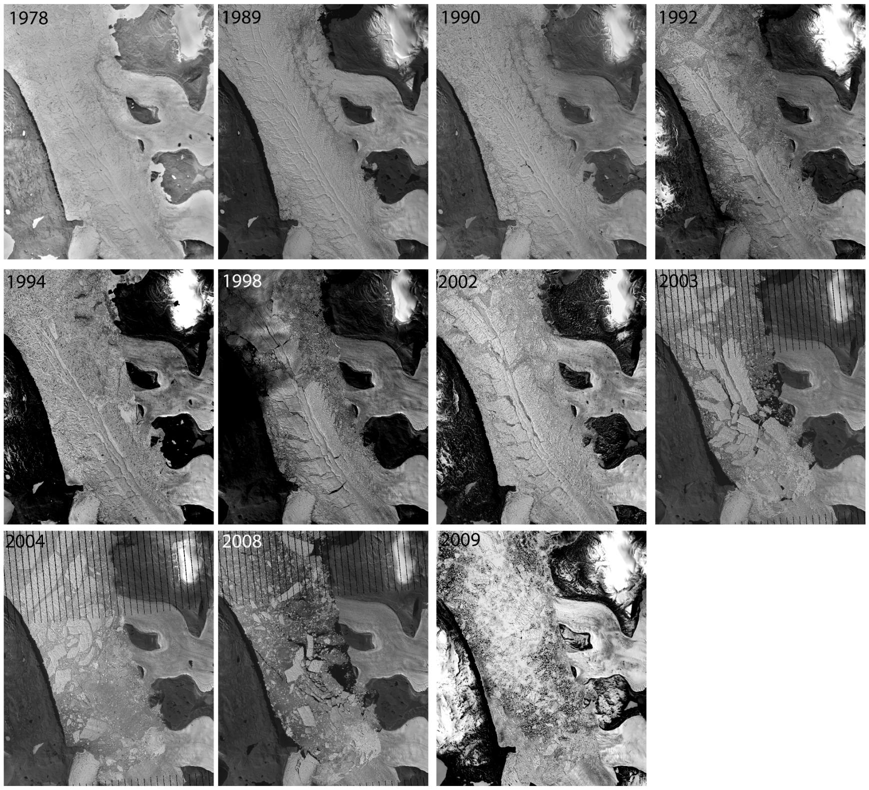

Fig. S1. Ostenfeld ice shelf collapse observed using optical imagery from the NASA/USGS Landsat satellites. Black stripes are due to the failure of the Scan Line Corrector in Landsat-7 data.

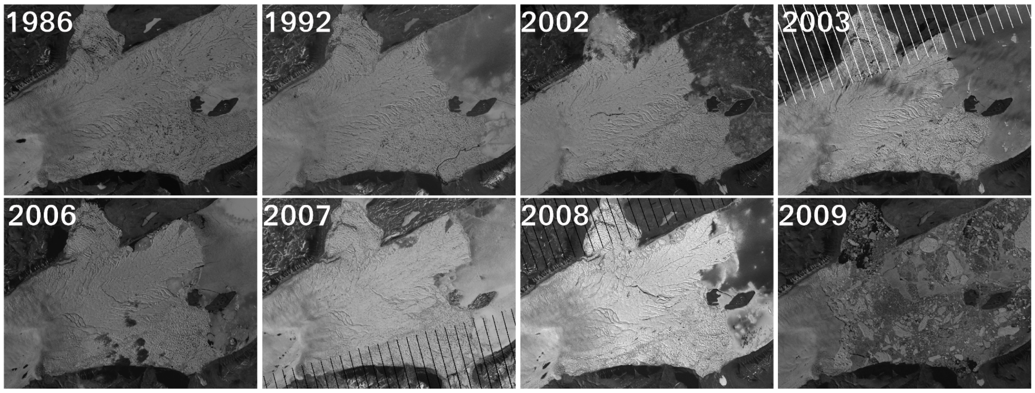

Fig. S2. Hagen Bræ ice shelf collapse observed using optical imagery from the NASA/USGS Landsat satellites.

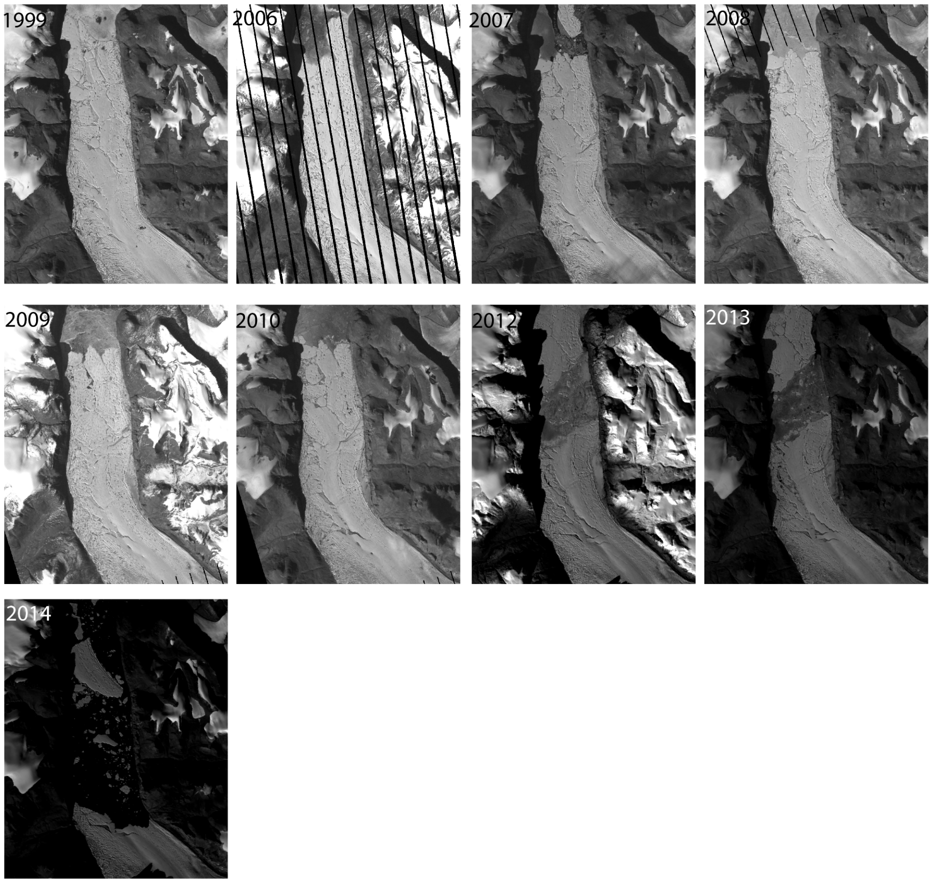

Fig. S3. Steensby glacier ice shelf collapse observed using optical imagery from the NASA/USGS Landsat satellites. Black stripes are due to the failure of the Scan Line Corrector in Landsat-7 data.

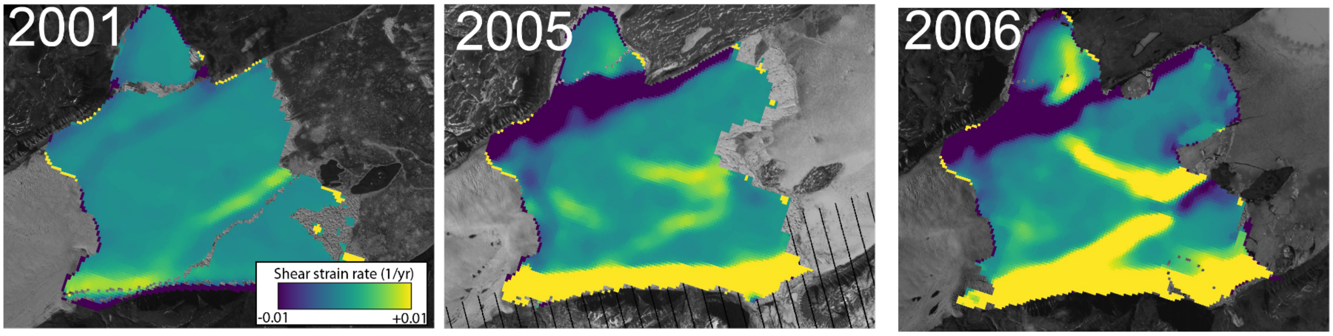

Fig. S4. Shear strain rates evolution over Hagen Bræ ice shelf, calculated using yearly surface flow velocity measurements between 2001 and 2007 (see Methods). Background images are Landsat

panchromatic images from the three respective years. Black stripes are due to the failure of the Scan Line Corrector in Landsat-7 data.

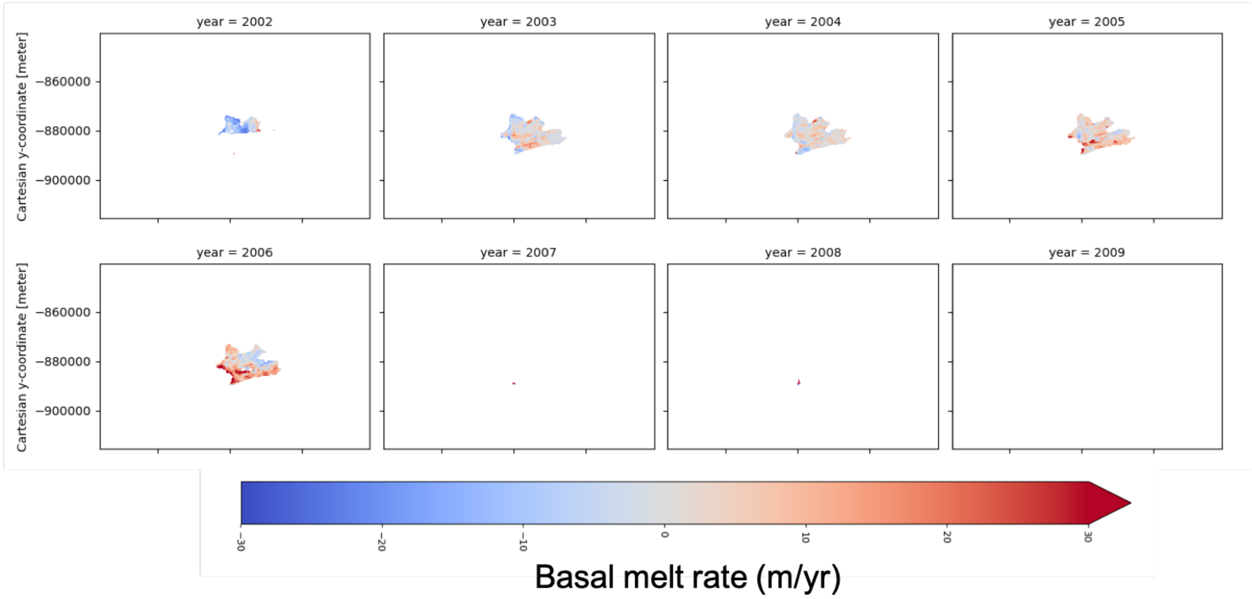

Fig. S5. Basal melting observed at the ice shelf of Hagen Bræ between 2002 and 2006.

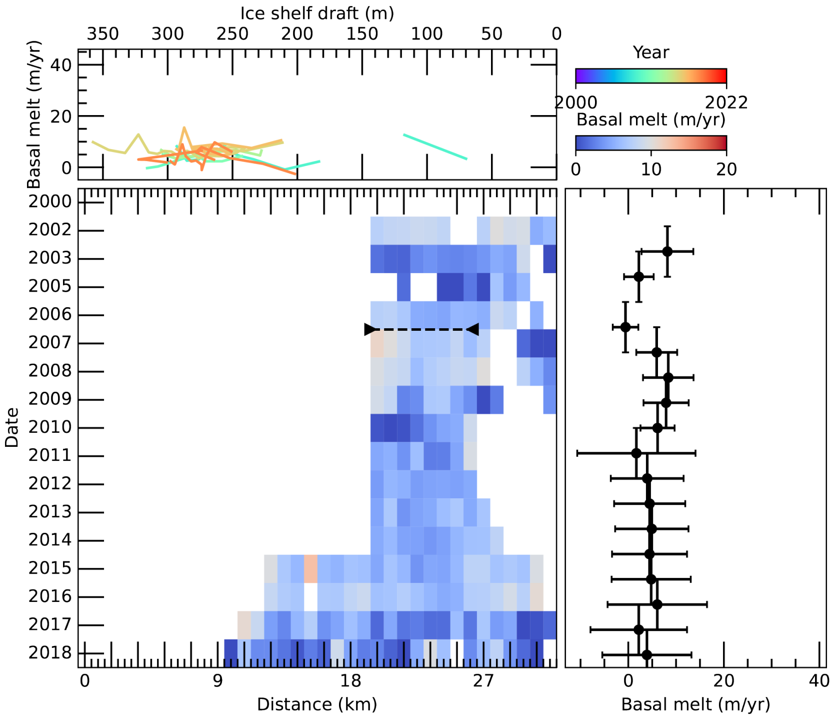

Fig. S6. Changes in basal melt rates over Størestrommen/Bistrup Bræ. Change in basal melt rate is represented as an hovmöller diagram along the ice shelf length, where basal melt rates are averaged along given cross section. Melt rate evolution are also provided as a function of the ice shelf draft. Averaged grounding line melt rate inside the black dotted line are also provided.

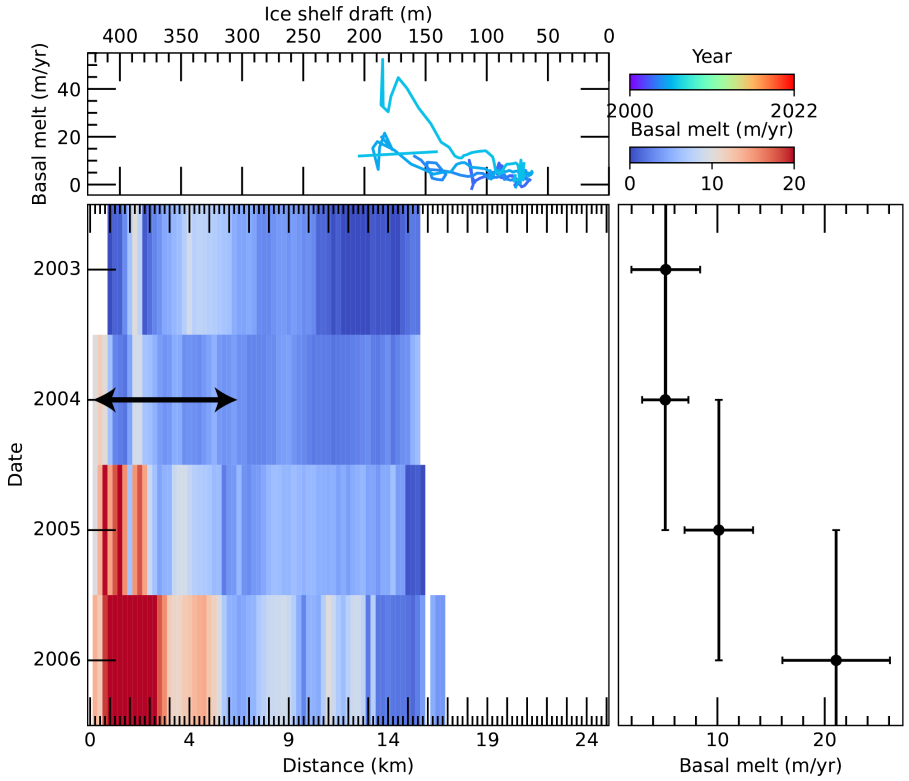

Fig. S7. Changes in basal melt rates over Hagen Bræ. Change in basal melt rate is represented as an hovmöller diagram along the ice shelf length, where basal melt rates are averaged along given cross section. Melt rate evolution are also provided as a function of the ice shelf draft. Averaged grounding line melt rate inside the black dotted line are also provided.

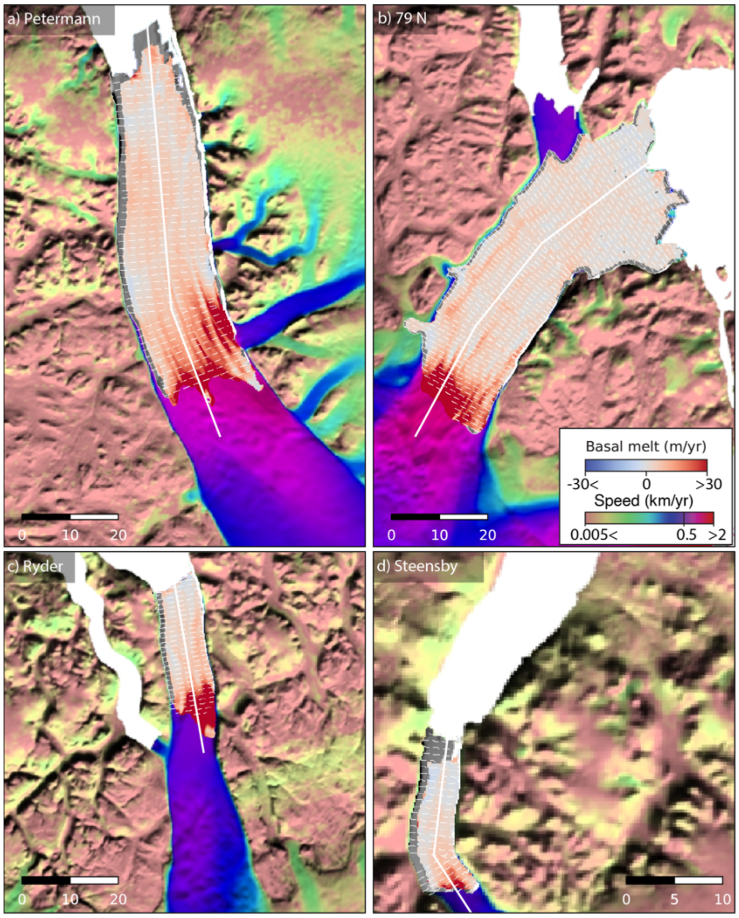

Fig. S8. Average basal melt rate map between 2000 and 2021 observed at (a) Petermann, (b) 79N, (c) Ryder and (d) Steensby glacier. The Fig. shows the position of the flowline and cross-section used to calculate width-averaged basal melt rates in Fig. 2. Surface flow velocity is shown on a logarithmic color scale outside of floating ice (source: [1]).

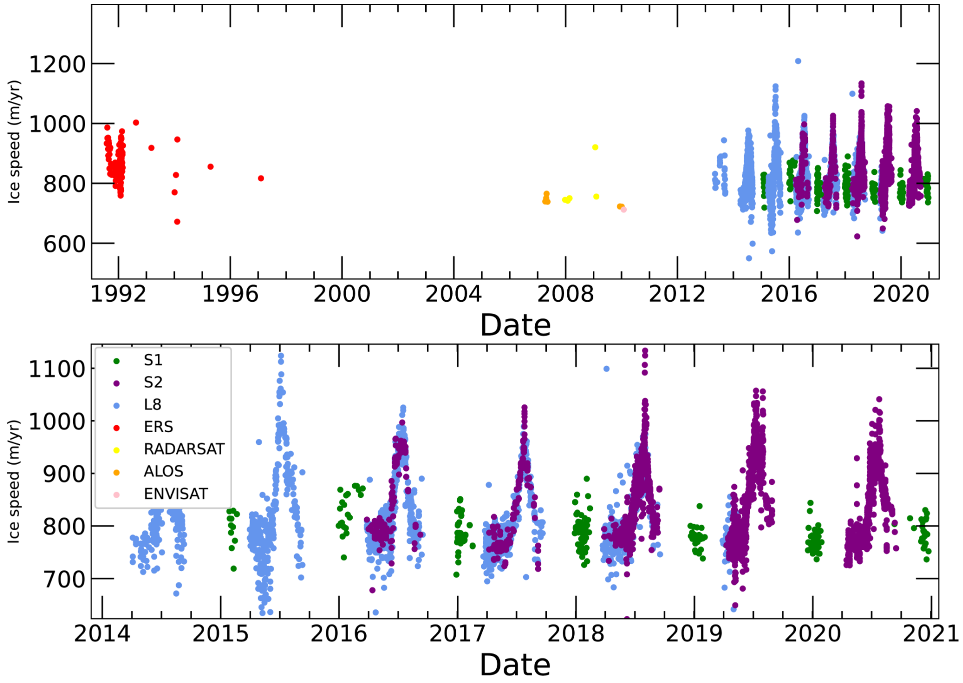

Fig. S9. Grounding line ice flow velocity of Ostenfeld glacier. The ice flow velocity is averaged inside a box located close the grounding line of the glacier.

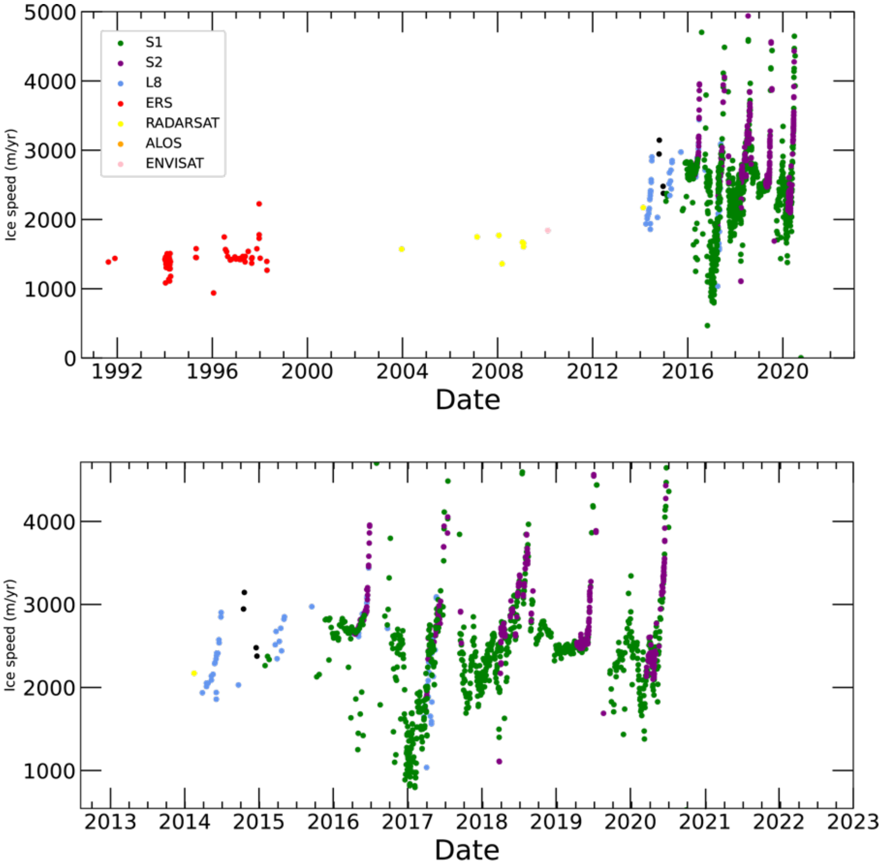

Fig. S10. Grounding line ice flow velocity of Zachariae glacier. The ice flow velocity is averaged inside a box located close the grounding line of the glacier.

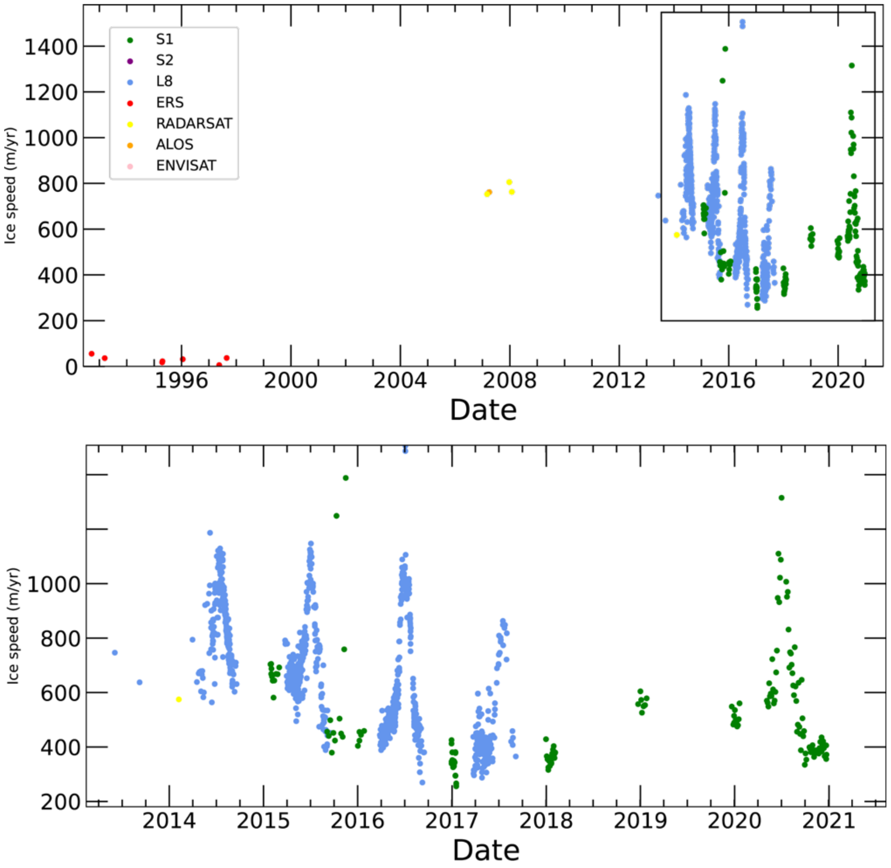

Fig. S11. Grounding line ice flow velocity of Hagen Bræ. The ice flow velocity is averaged inside a box located close the grounding line of the glacier.

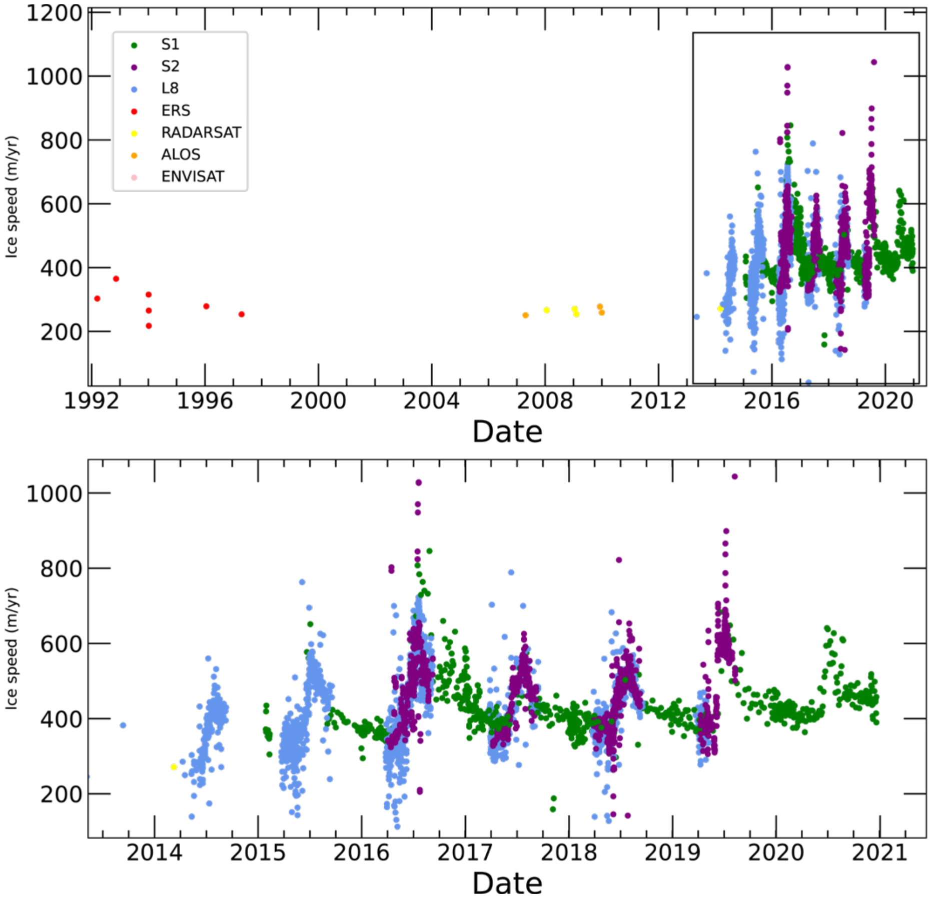

Fig. S12. Grounding line ice flow velocity of Steensby. The ice flow velocity is averaged inside a box located close the grounding line of the glacier.

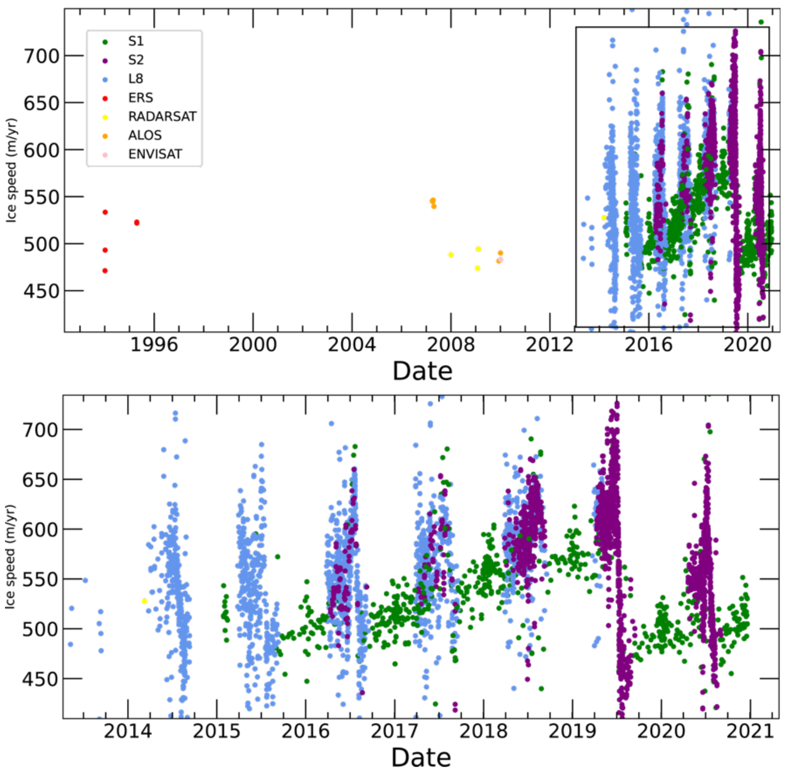

Fig. S13. Grounding line ice flow velocity of Ryder glacier.

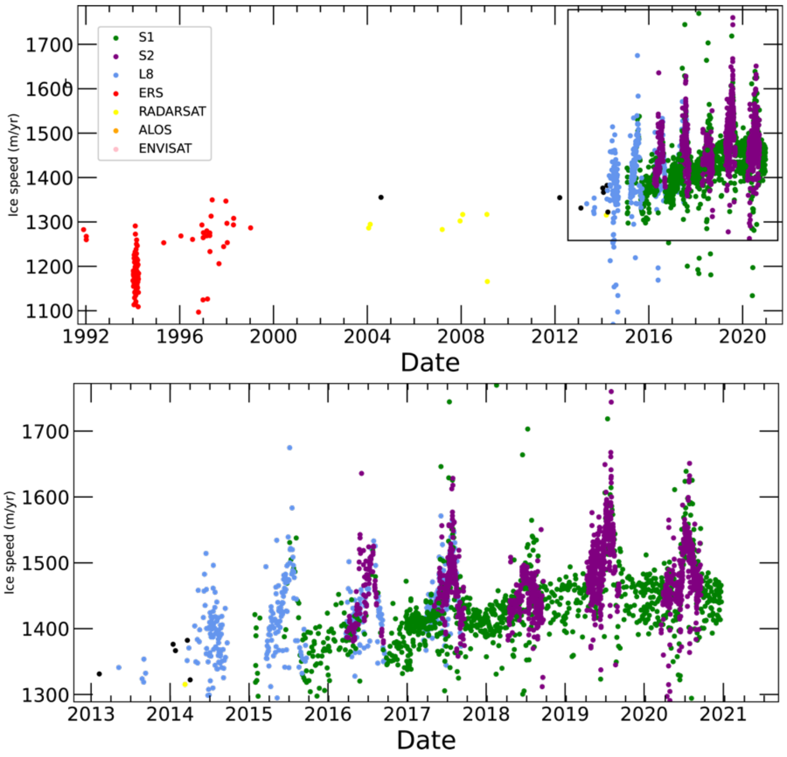

Fig. S14. Grounding line ice flow velocity of 79N glacier. The ice flow velocity is averaged inside a box located close to the grounding line of the glacier.

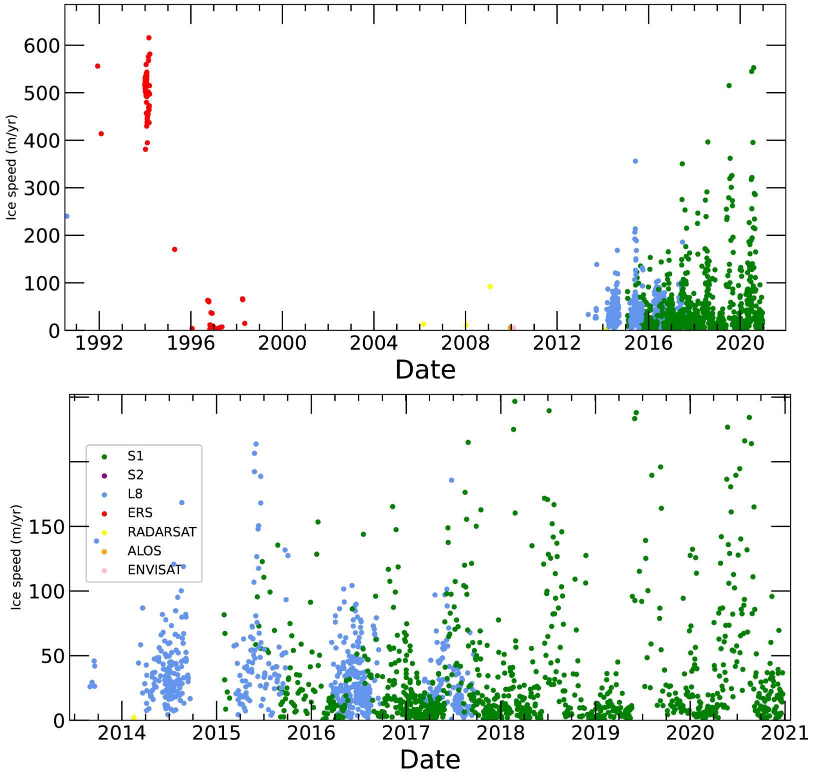

Fig. S15. Grounding line ice flow velocity of Bistrup Bræ glacier. The ice flow velocity is averaged inside a box located close to the grounding line of the glacier.

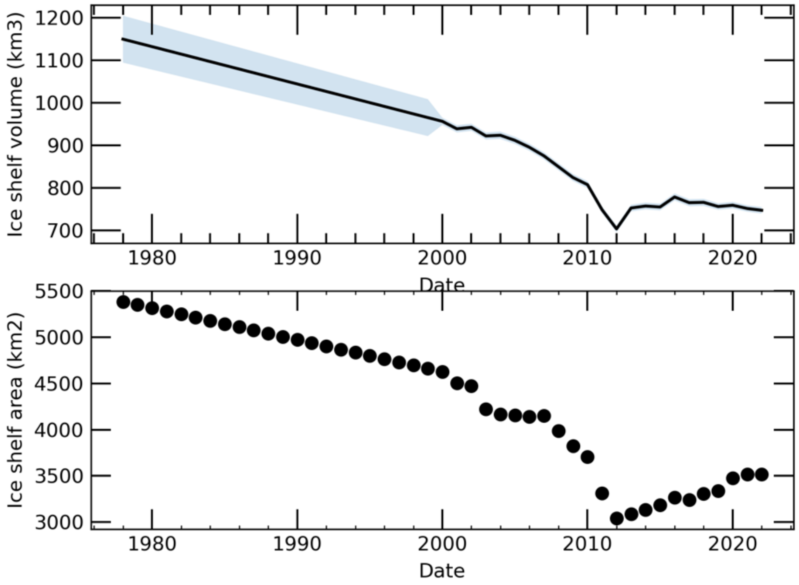

Fig. S16. Total ice shelf volume (top) and area (bottom) changes observed on the period 1978 - 2022.

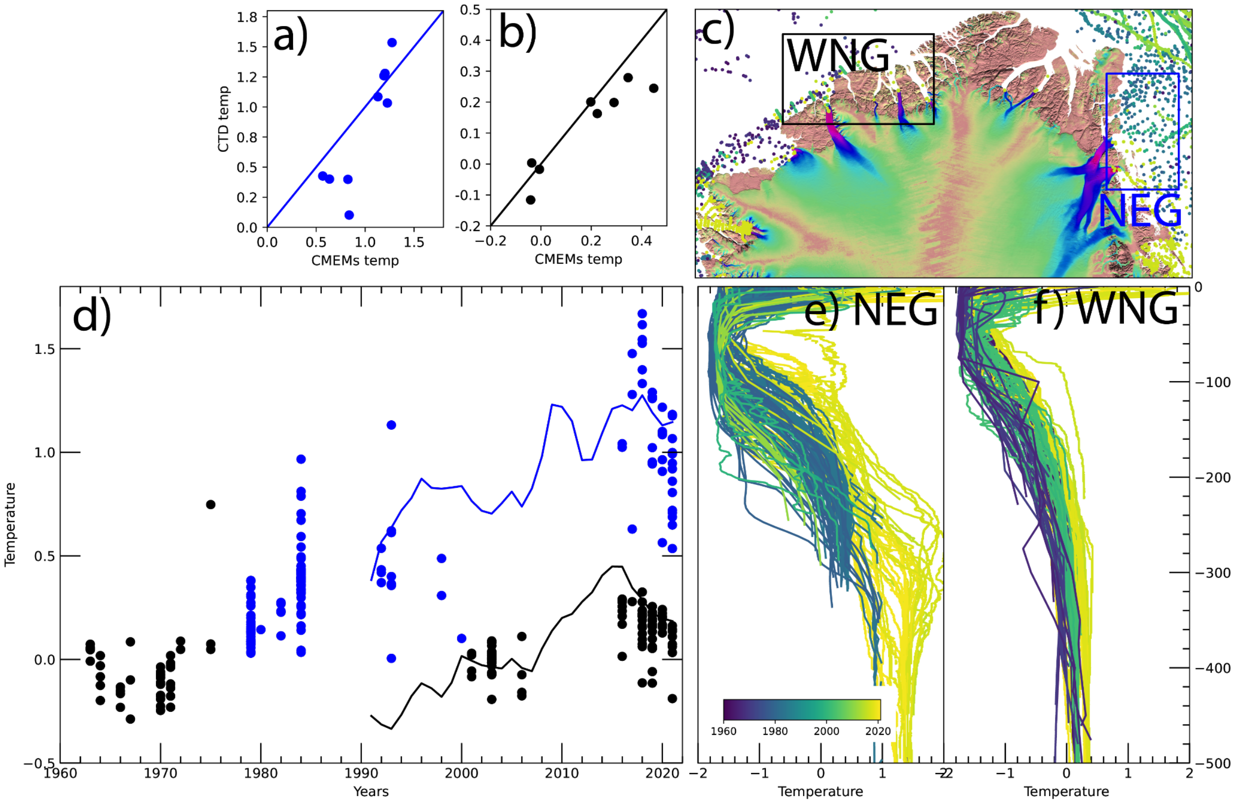

Fig. S17. Ocean conditions from in-situ CTD measurement and ocean physics reanalysis data. a) and b) are showing the fit between in-situ and data reanalysis, c) is showing the location of the CTD measurements across North and Northeast Greenland with the bounding box location used to calculate average potential temperature. The ice flow velocity of the Greenland ice sheet is shown on a logarithmic color scale (source:[1]). (d). (e) and (f) show the CTD temperature profiles within each bounding box.

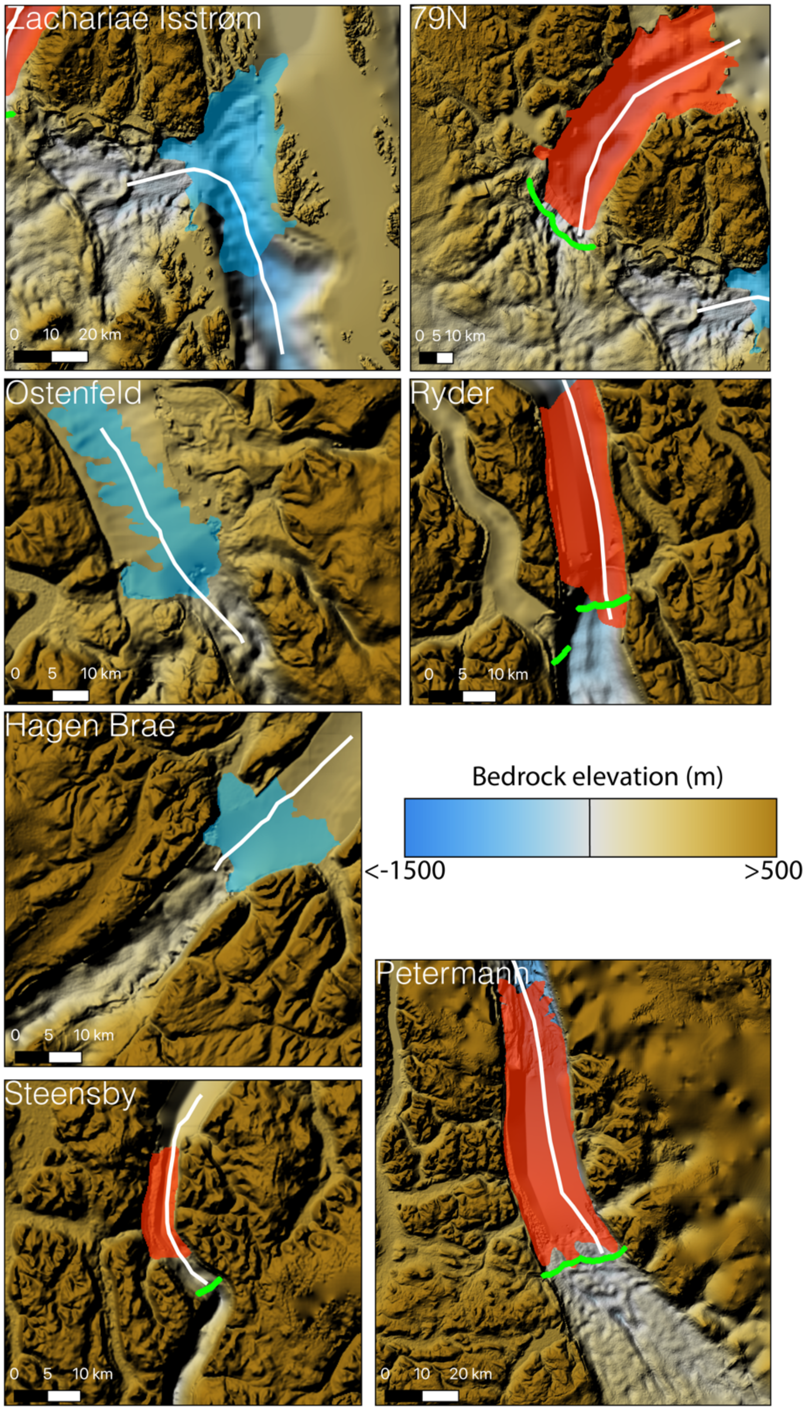
Zachariae Isstrøm
0 10 20 km
79N
0 5 10 km
Ostenfeld
0 5 10 km
Ryder
0 5 10 km
Hagen Brae
0 5 10 km
Bedrock elevation (m)
<-1500
>500
Petermann
0 10 20 km
Steensby
0 5 10 km

Fig. S18. Bedrock and bathymetry of the glaciers and ice shelves of North Greenland. The bedrock elevation source is from BedMachine v3[2]. Ice shelves that have collapsed are shown in blue color, and still standing ice shelves are represented in red. The white line shows the flowline profiles that are used in Fig S14-S19.

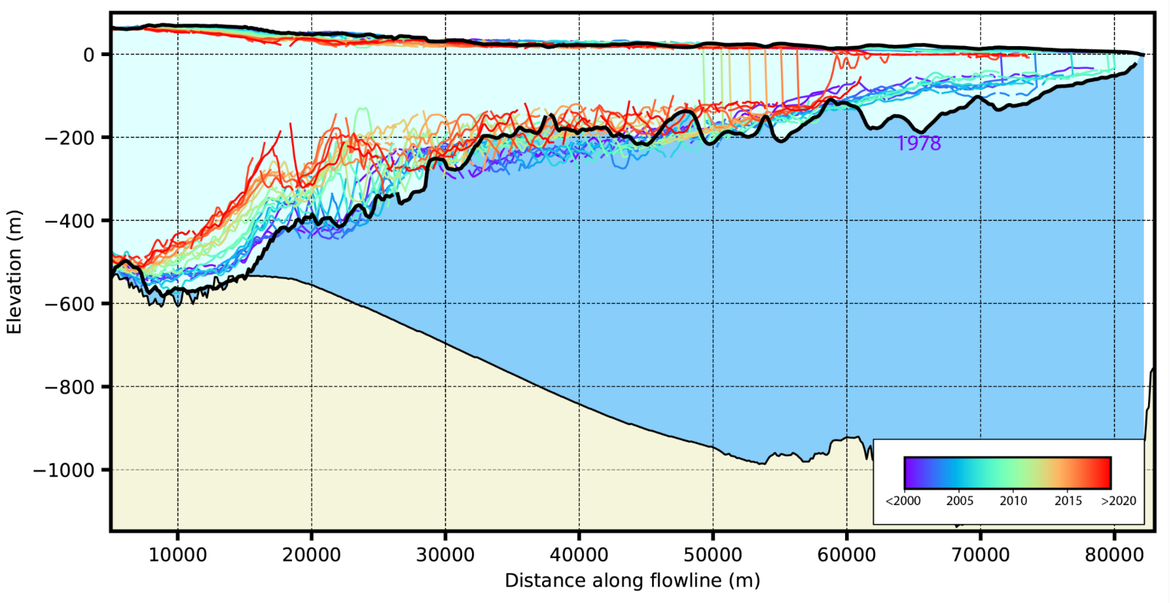

Fig. S19. Thinning of Petermann ice shelf along flowline between 1978 and present (see Fig S13).

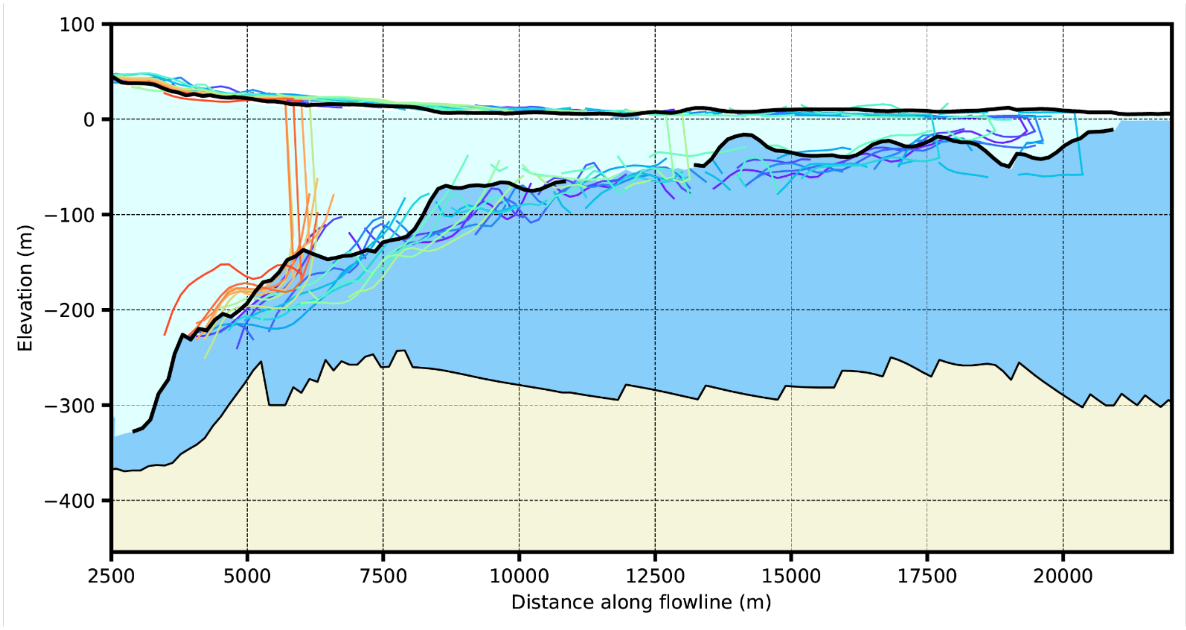

Fig. S20. Thinning of Steensby ice shelf along flowline between 2001 and present (see Fig S13).

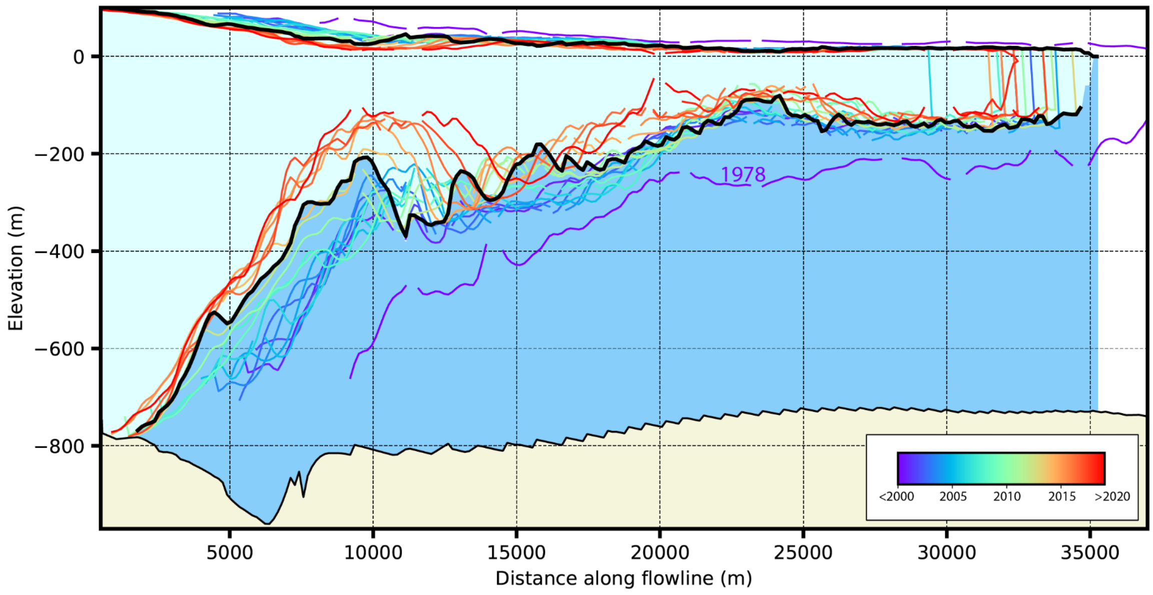

Fig. S21. Thinning of Ryder ice shelf along flowline between 1978 and present (see Fig S13).

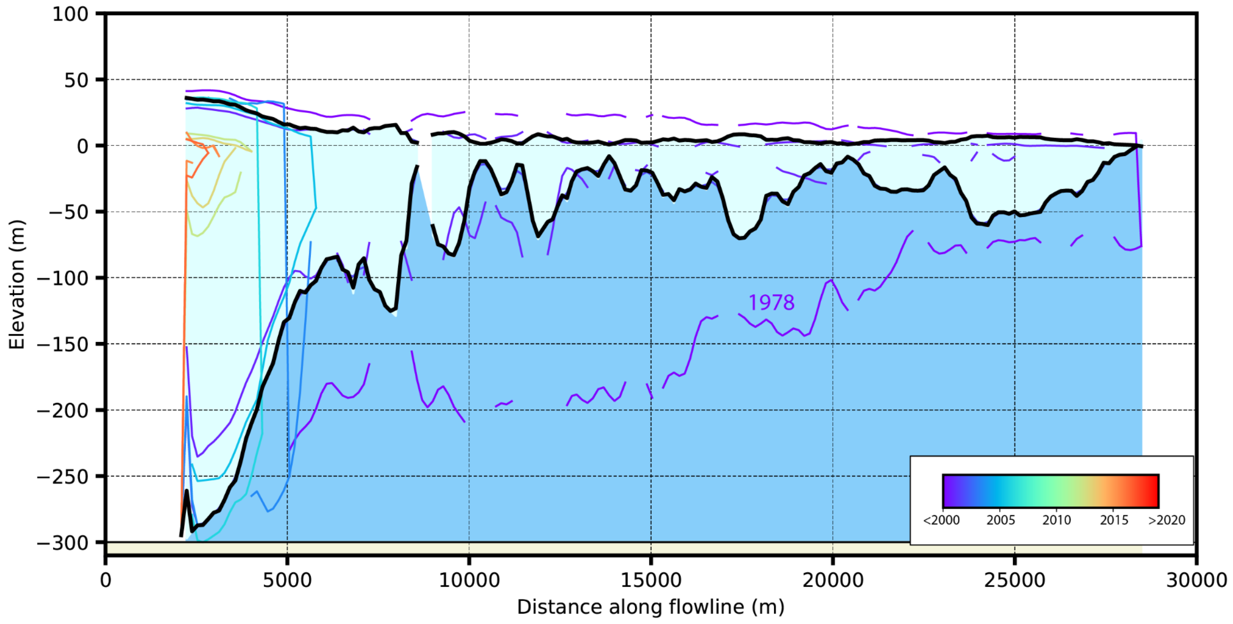

Fig. S22. Thinning and collapse of Ostenfeld ice shelf along flowline between 1978 and present (see Fig S13)

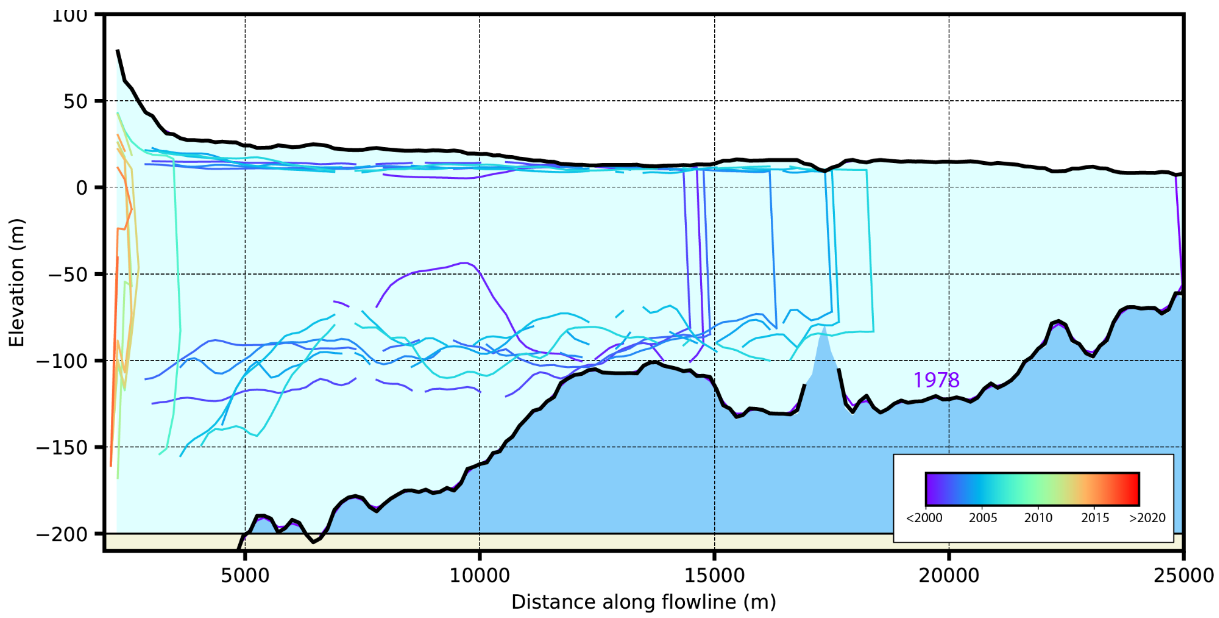

Fig. S23. Thinning and collapse of Hagen Brae ice shelf along flowline between 1978 and present (see Fig S13)

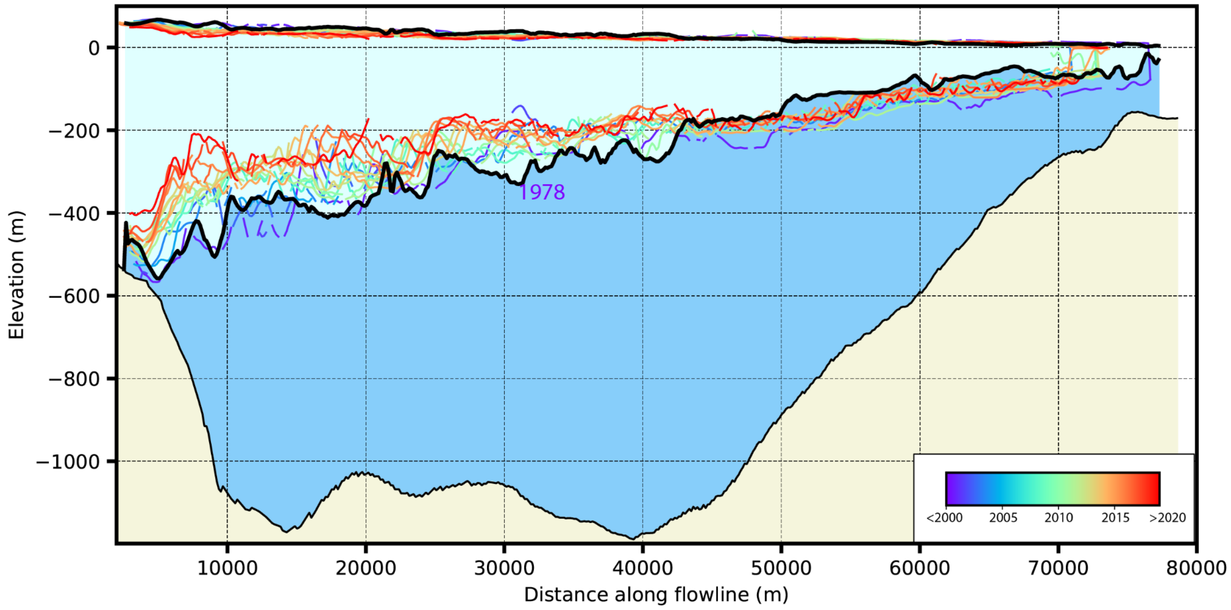

Fig. S24. Thinning of 79N ice shelf along flowline between 1978 and present (see Fig S13).

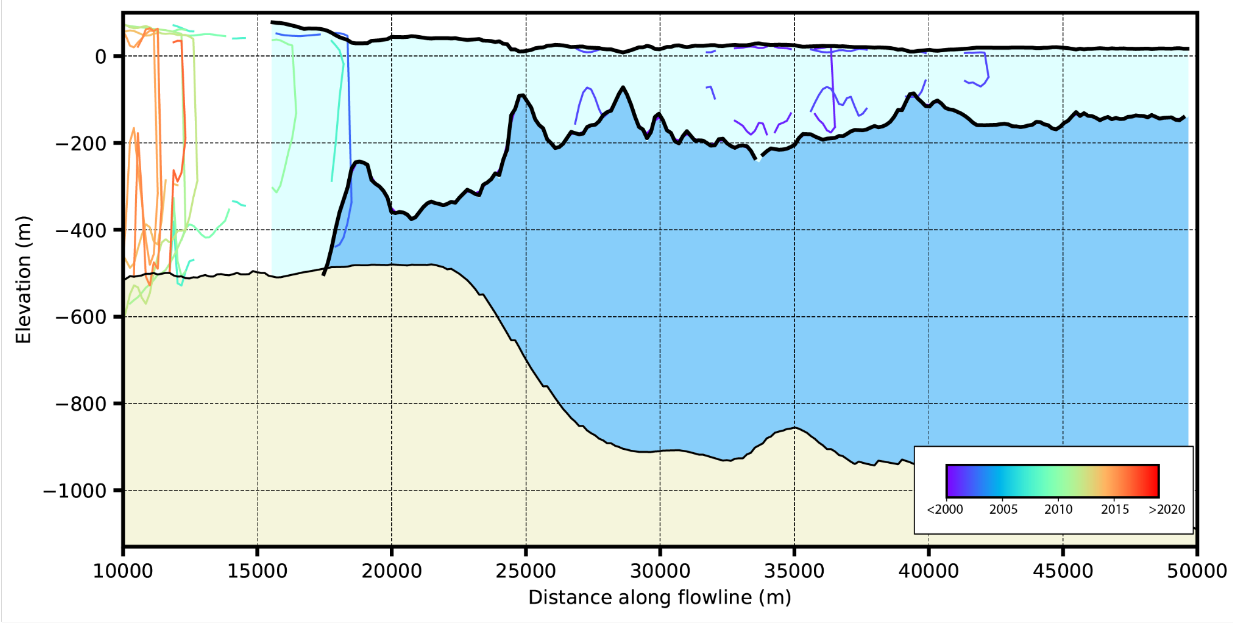

Fig. S25. Thinning and collapse of Zachariæ Isstrøm along flowline between 1978 and present (see Fig S13).

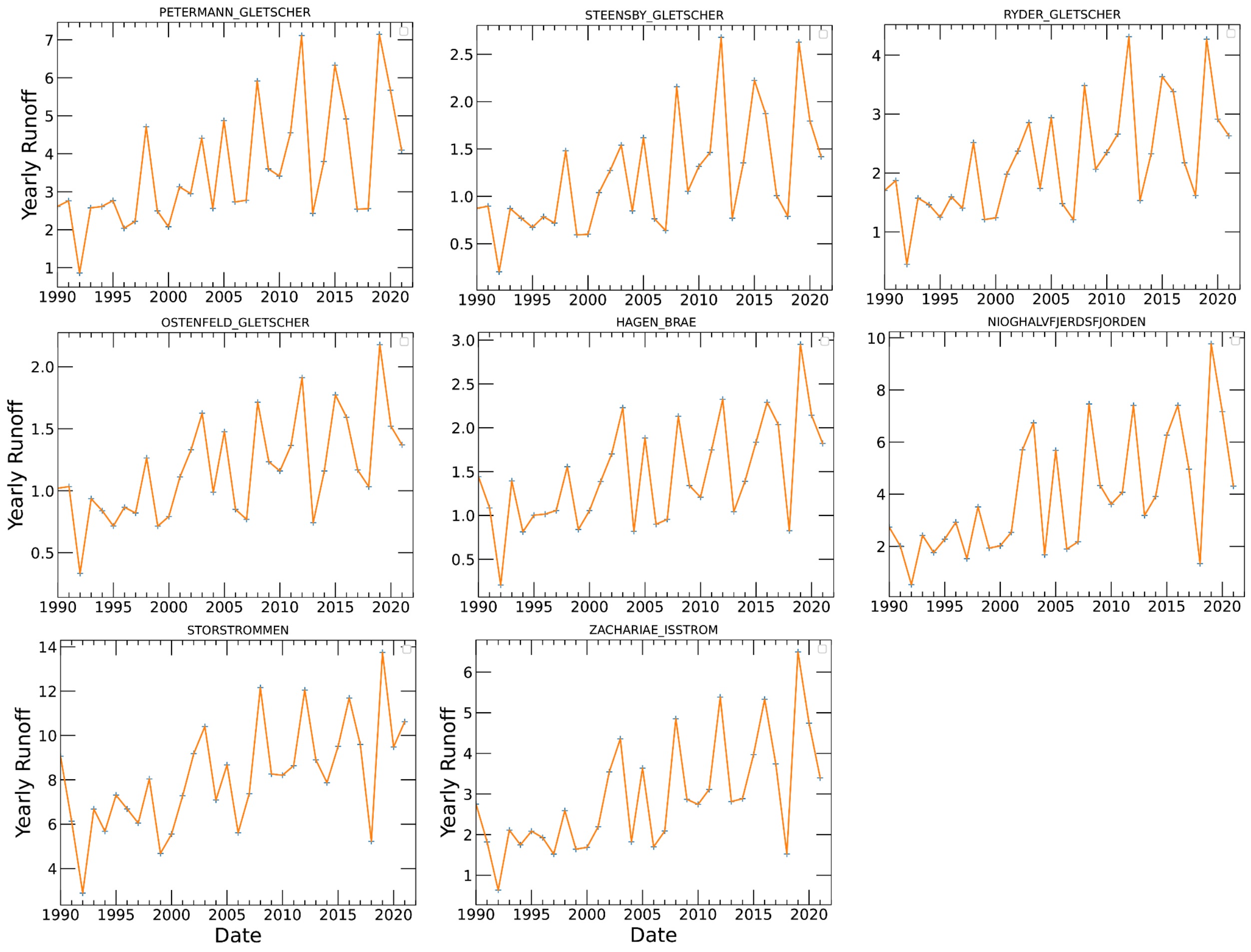

Fig. S26. Yearly evolution of the runoff extracted from the MAR model output over each glacier drainage basin[1].

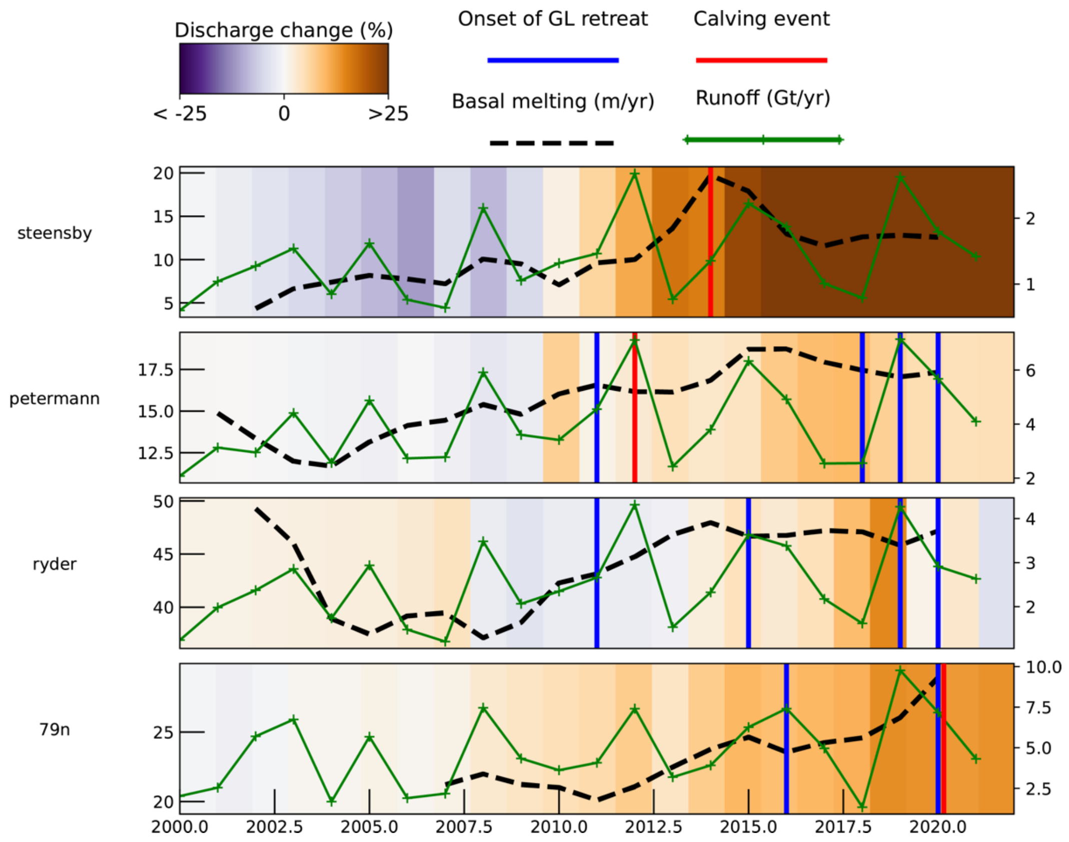

Fig. S27. Glaciers dynamical response to ice shelves changes (a-d). Changes in ice discharge is represented in % relative to the average of 1970-2000, and color coded from blue to red. Ice shelf melt rate evolution is represented as black dotted line. Calving events and onset of GL retreat are noted as vertical blue and yellow bars respectively. e) Changes in runoff for each glacier drainage basin is represented in green.

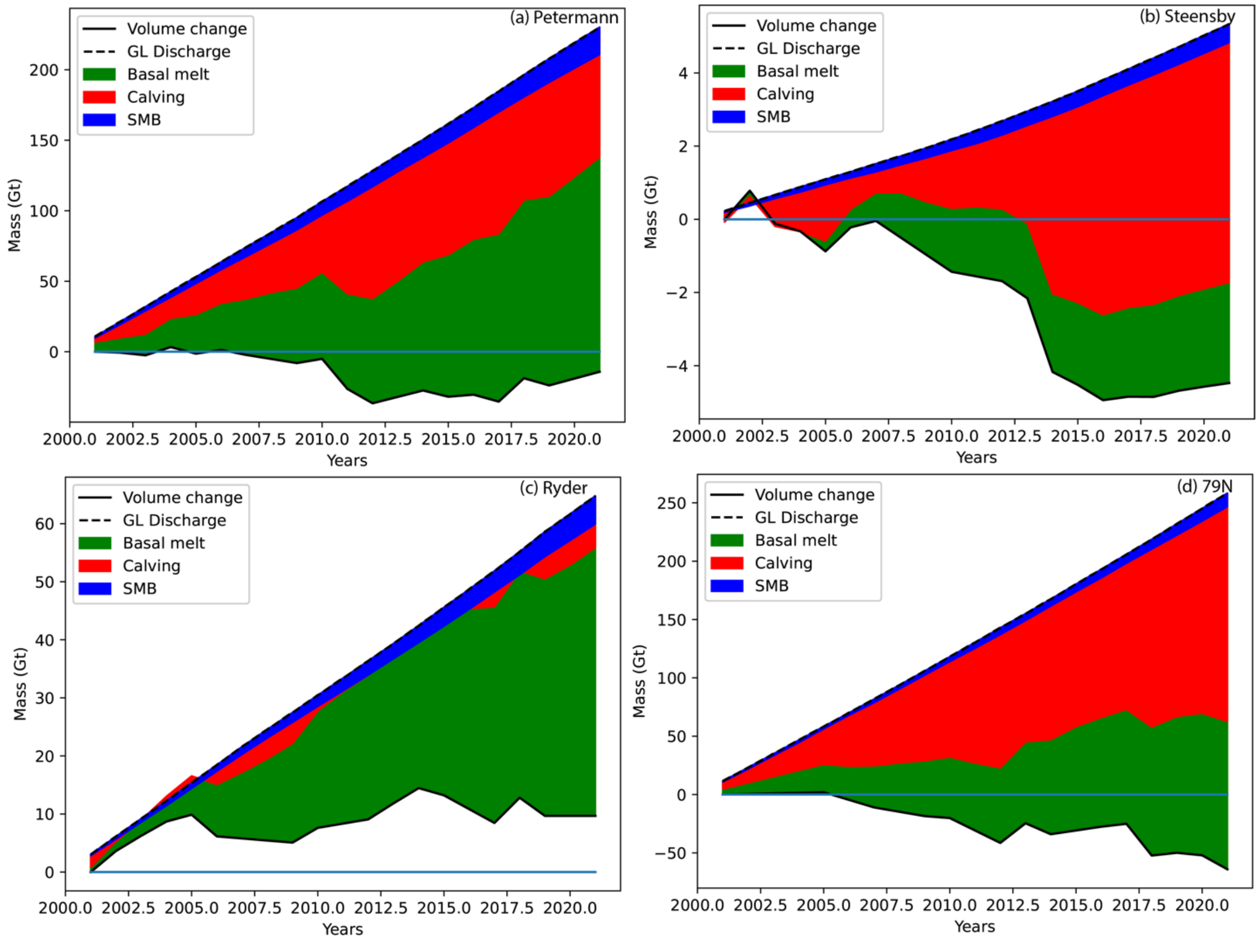

Fig S28. Ice shelf mass balance partitioning evolution for Petermann (a), Steensby (b), Ryder (c) and 79N (d).

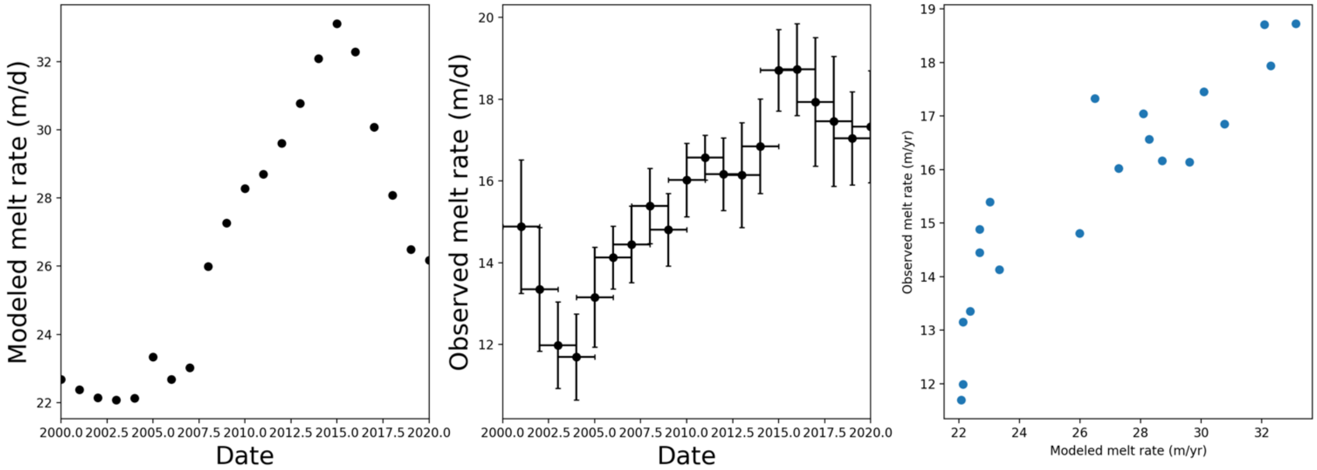

Fig S29. Modeled melt rate using the parametrization from[26] and neglecting subglacial water discharge (left), observed melt rates (center) and comparison between the two calculations (right).

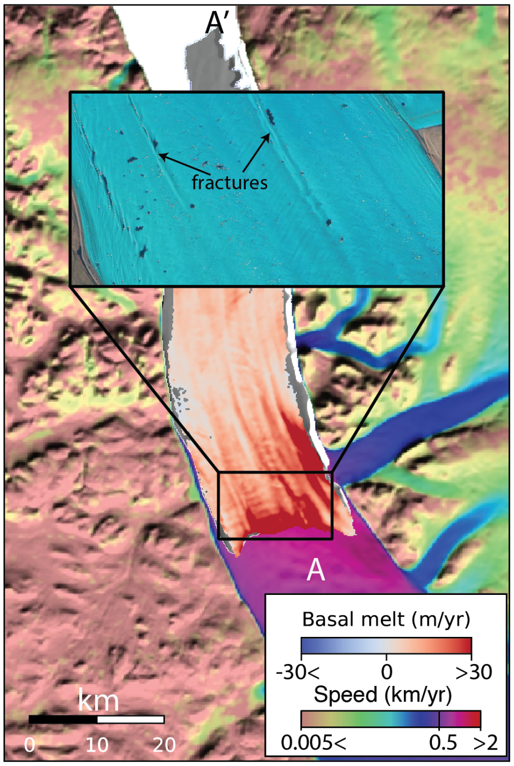

Fig S30. Basal melt rate averaged after 2015 over the Petermann ice shelf. The inset shows a Landsat-8 optical image from 2019 with a zoom at the grounding line highlighting the presence of large along flow fractures. The surface velocity map outside of grounded ice is from [1].

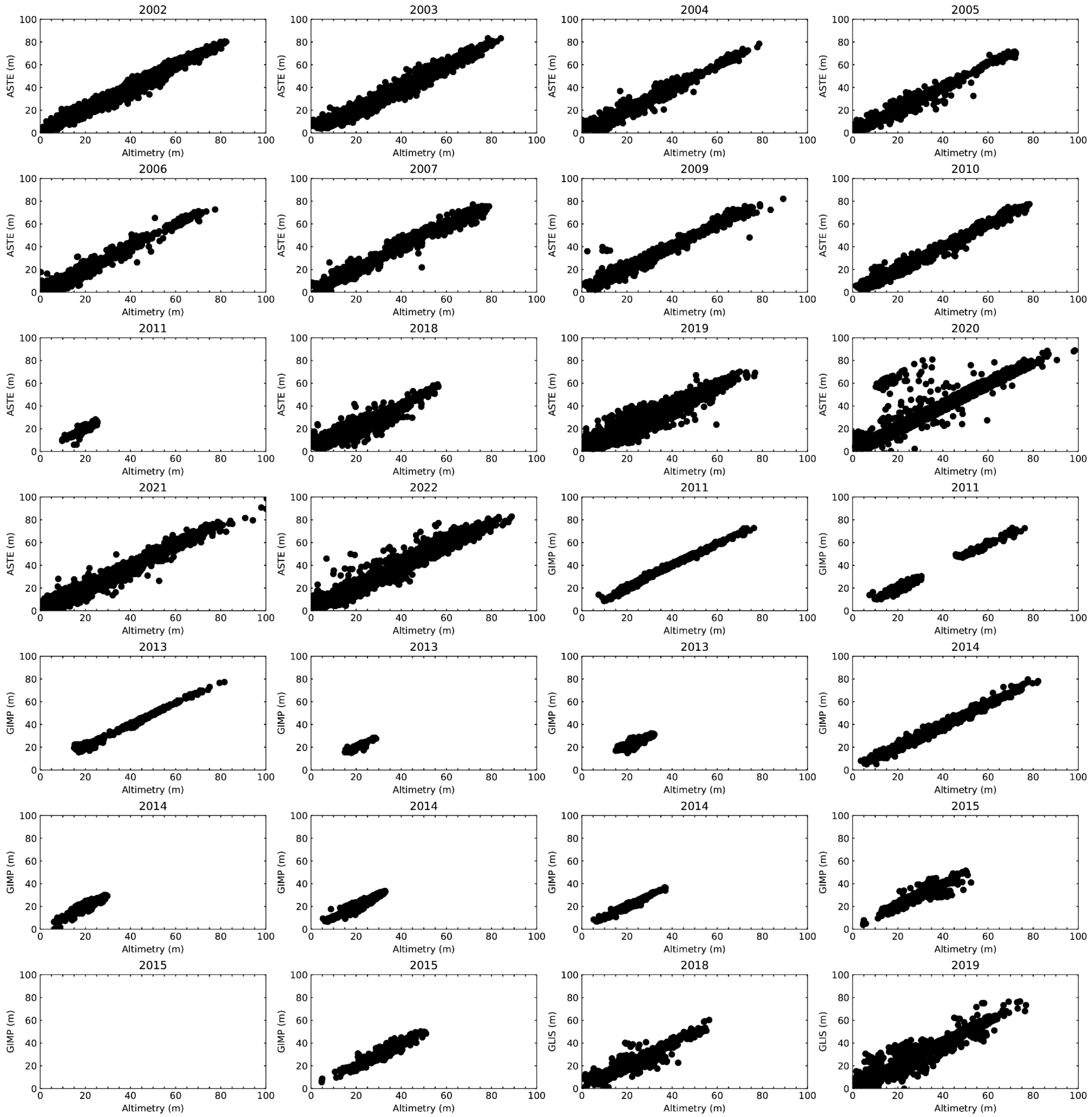

Fig. S31. Fit in surface elevation between ASTER (ASTE), GIMP and GLISTIN-A (GLIS) DEMs and yearly reference DEMs assembled using a combination of Altimetry data (see Material and Methods) over the Petermann ice shelf. Empty plot corresponds to years without overlap with altimetry data.

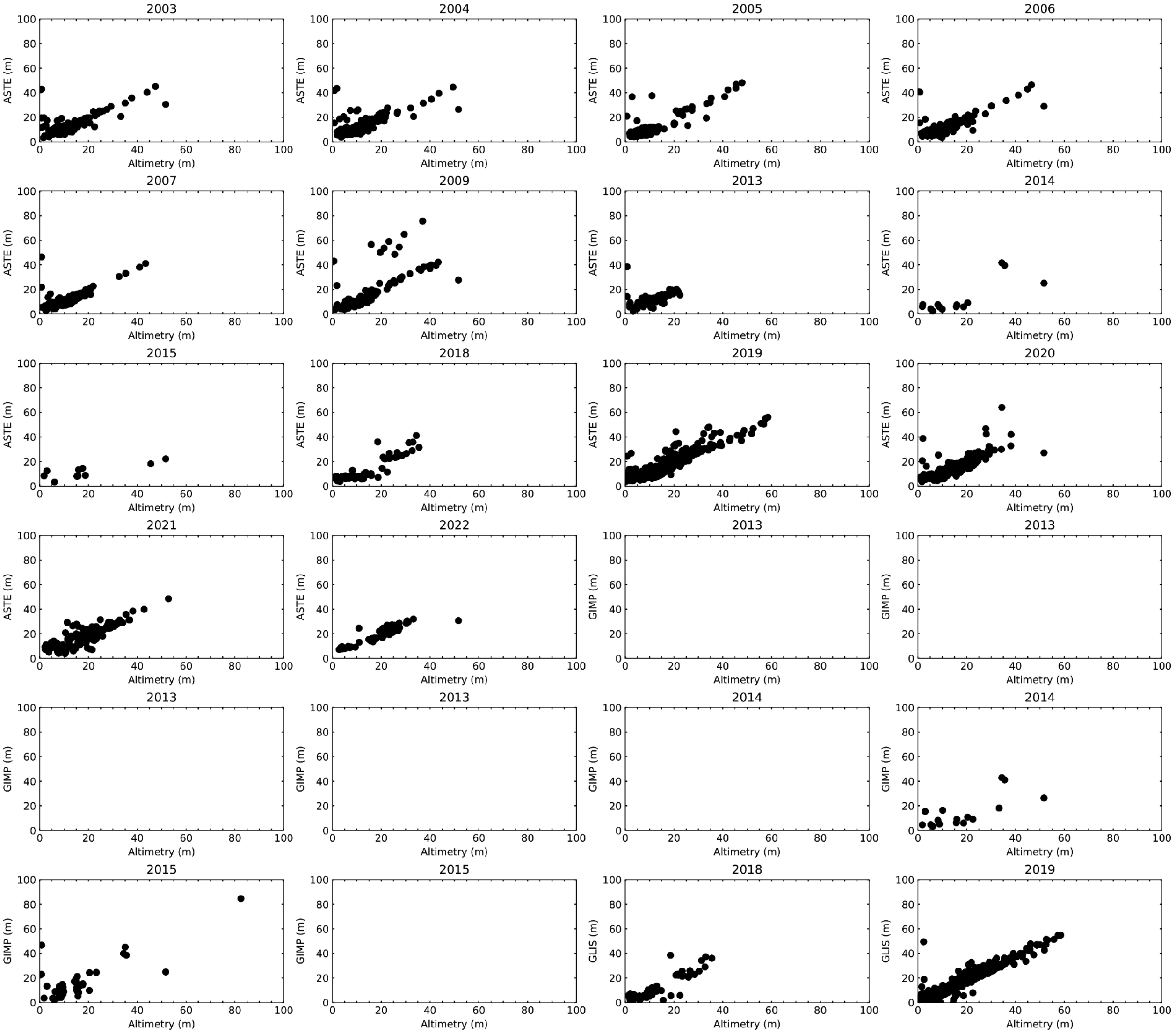

Fig. S32. Fit in surface elevation between ASTER (ASTE), GIMP and GLISTIN-A (GLIS) DEMs and yearly reference DEMs assembled using a combination of Altimetry data (see Material and Methods) over the Steensby ice shelf. Empty plot corresponds to years without overlap with altimetry data.

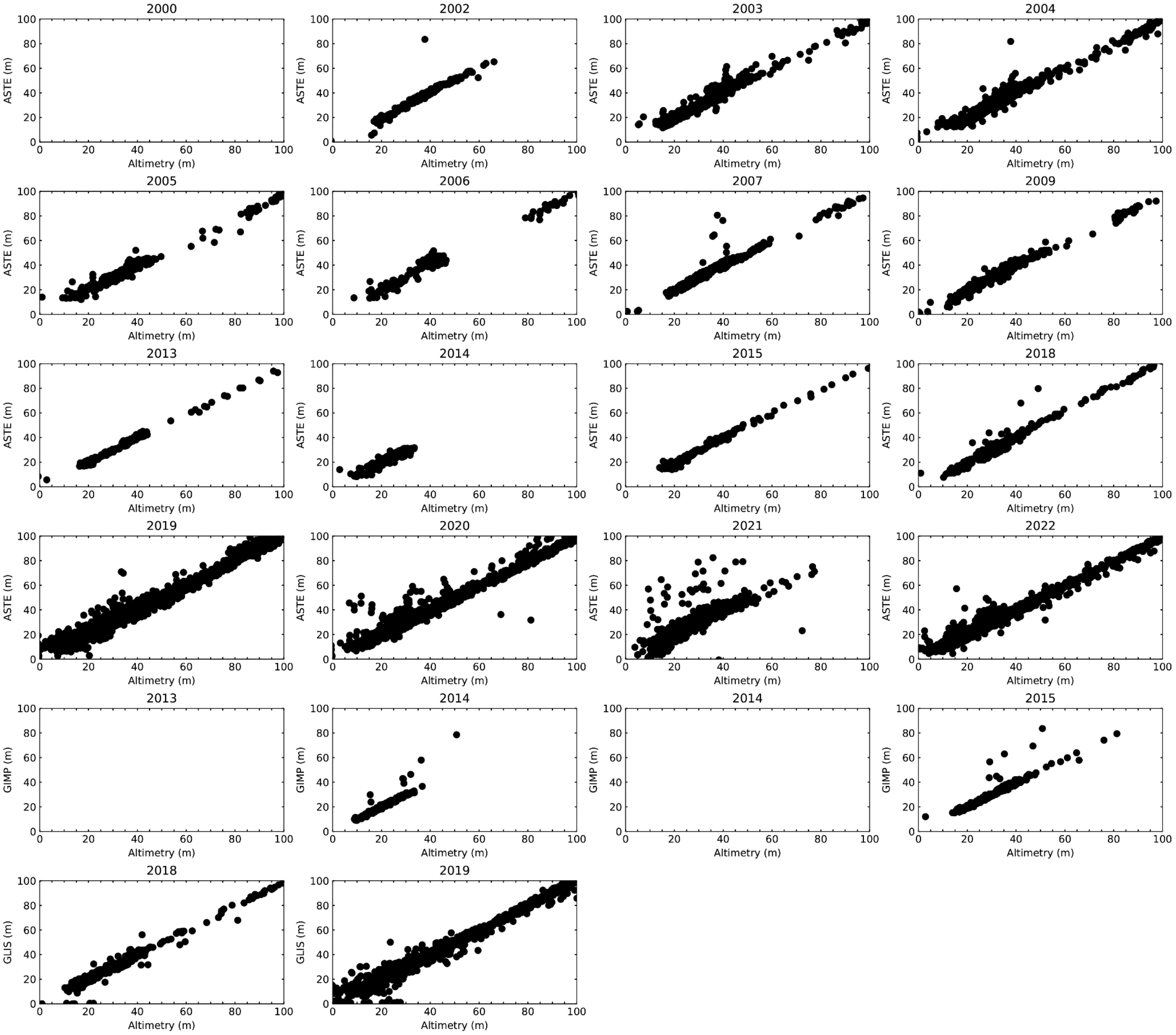

Fig. S33. Fit in surface elevation between ASTER (ASTE), GIMP and GLISTIN-A (GLIS) DEMs and yearly reference DEMs assembled using a combination of Altimetry data (see Material and Methods) over the Ryder ice shelf. Empty plot corresponds to years without overlap with altimetry data.

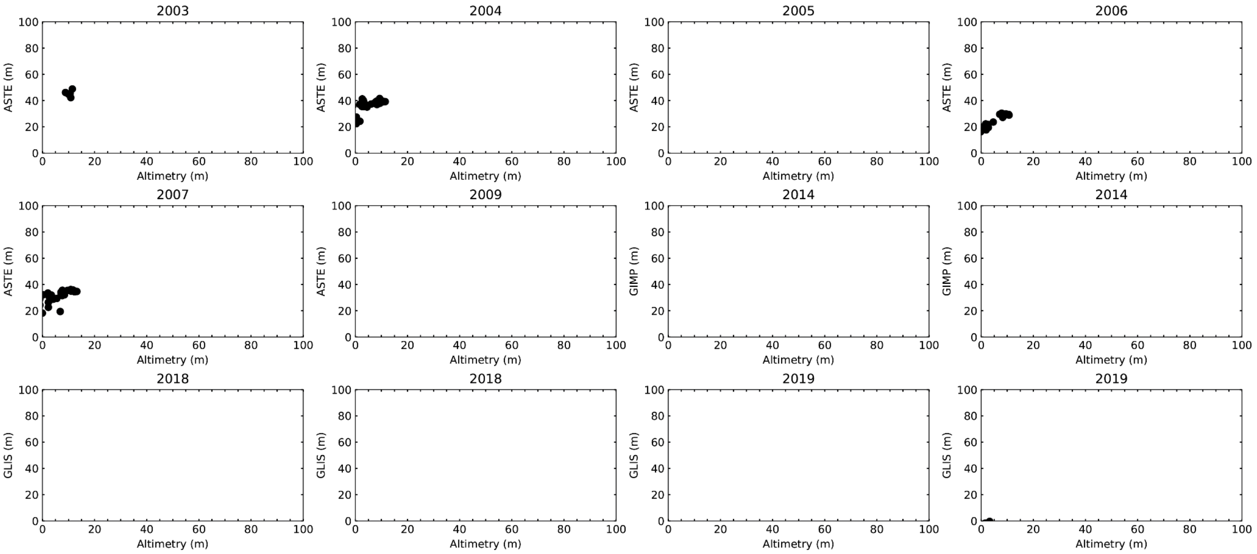

Fig. S34. Fit in surface elevation between ASTER (ASTE), GIMP and GLISTIN-A (GLIS) DEMs and yearly reference DEMs assembled using a combination of Altimetry data (see Material and Methods) over the Ostenfeld ice shelf. Empty plot corresponds to years without overlap with altimetry data.

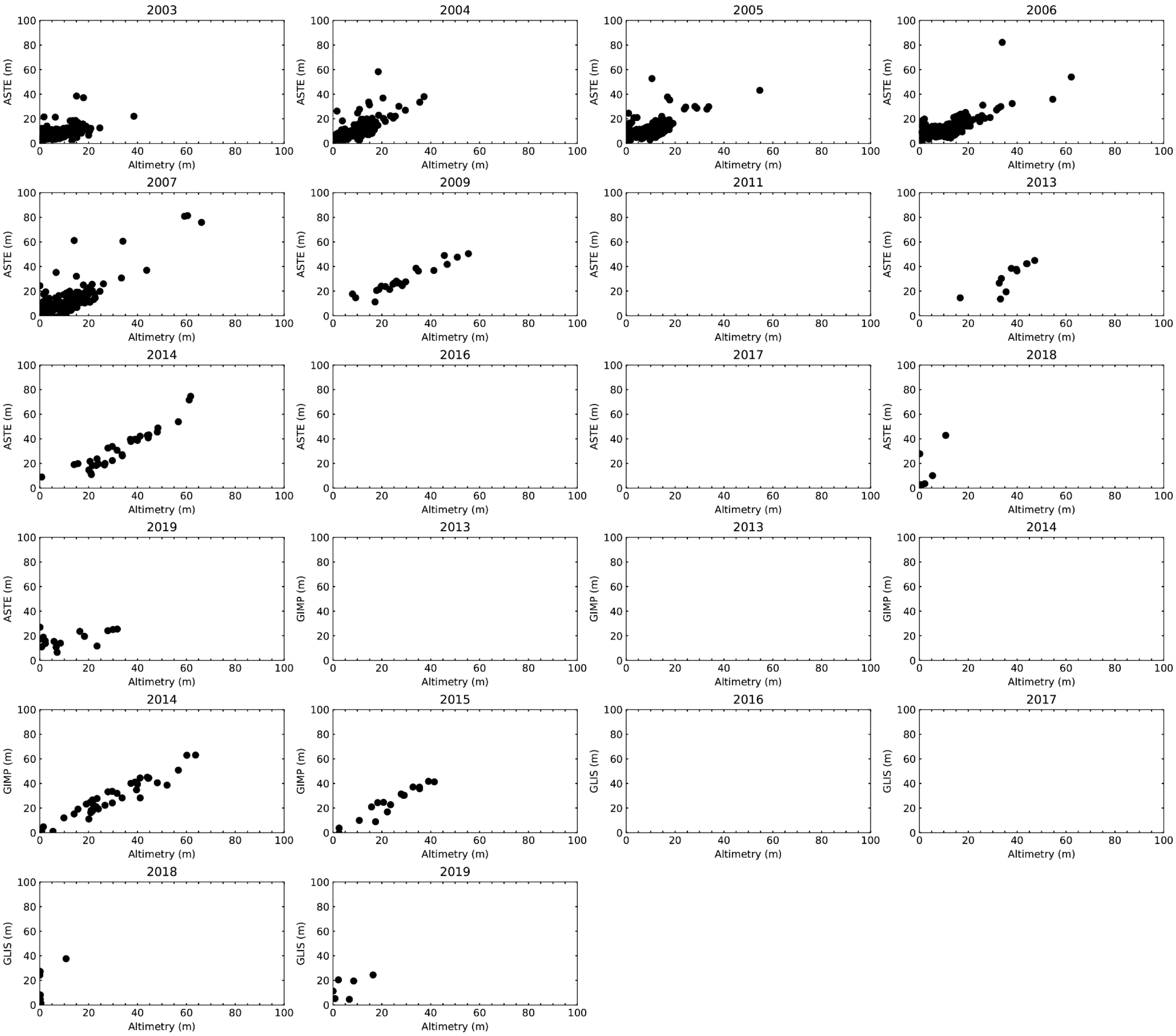

Fig. S35. Fit in surface elevation between ASTER (ASTE), GIMP and GLISTIN-A (GLIS) DEMs and yearly reference DEMs assembled using a combination of Altimetry data (see Material and Methods) over the Hagen Brae ice shelf. Empty plot corresponds to years without overlap with altimetry data.

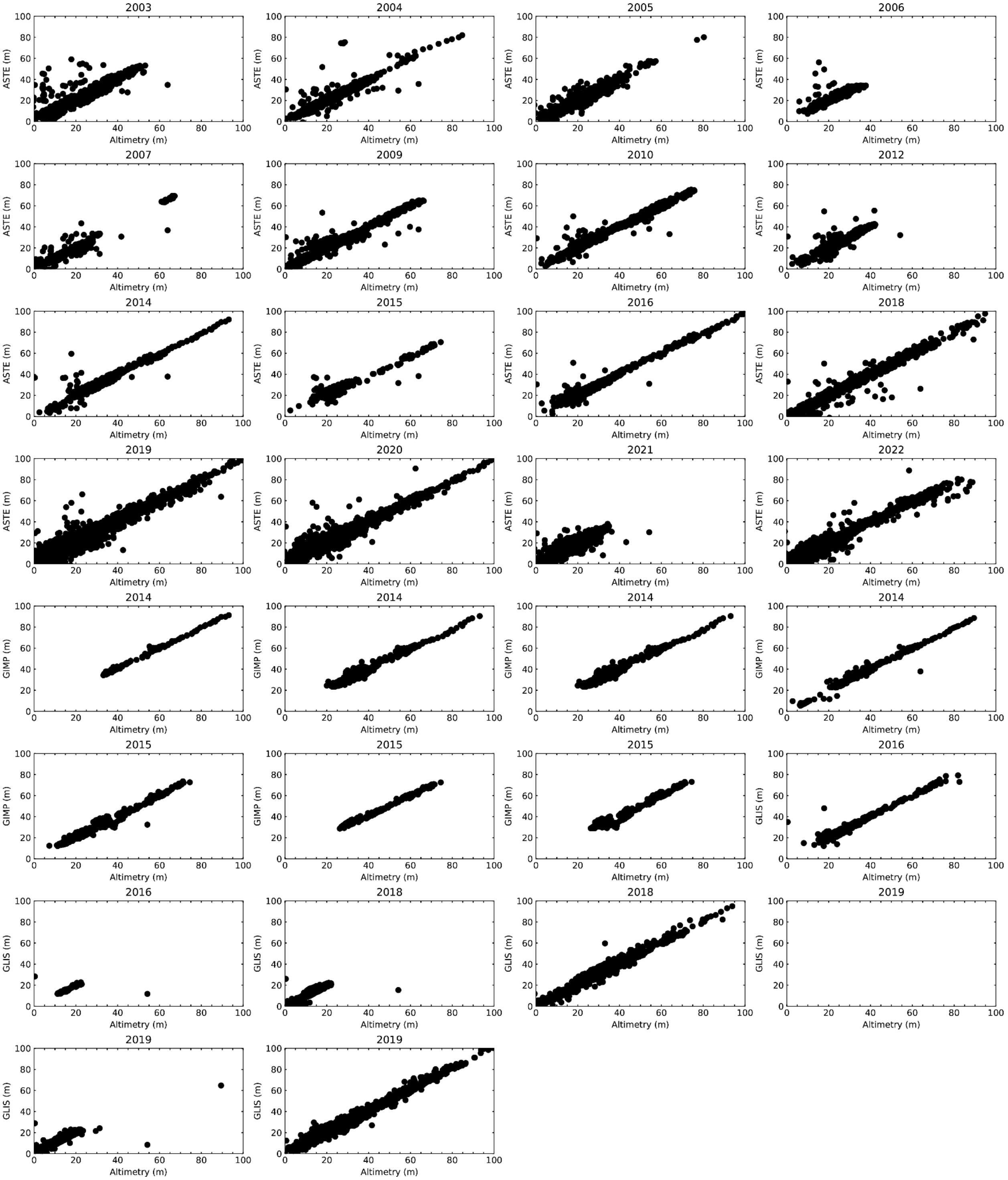

Fig. S36. Fit in surface elevation between ASTER (ASTE), GIMP and GLISTIN-A (GLIS) DEMs and yearly reference DEMs assembled using a combination of Altimetry data (see Material and Methods) over the 79N ice shelf. Empty plot corresponds to years without overlap with altimetry data.

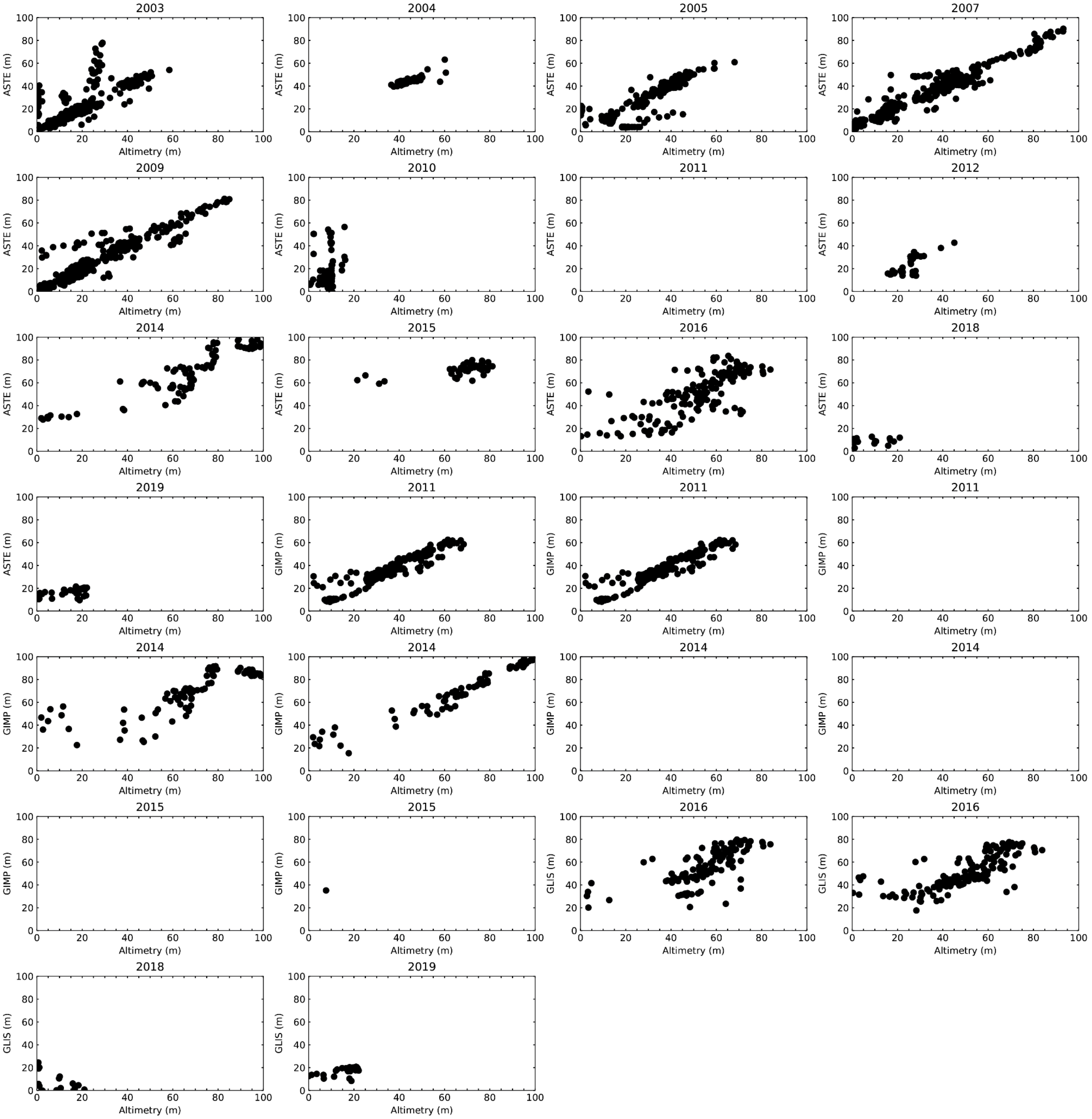

Fig. S37. Fit in surface elevation between ASTER (ASTE), GIMP and GLISTIN-A (GLIS) DEMs and yearly reference DEMs assembled using a combination of Altimetry data (see Material and Methods) over the Zachariae ice shelf. Empty plot corresponds to years without overlap with altimetry data.

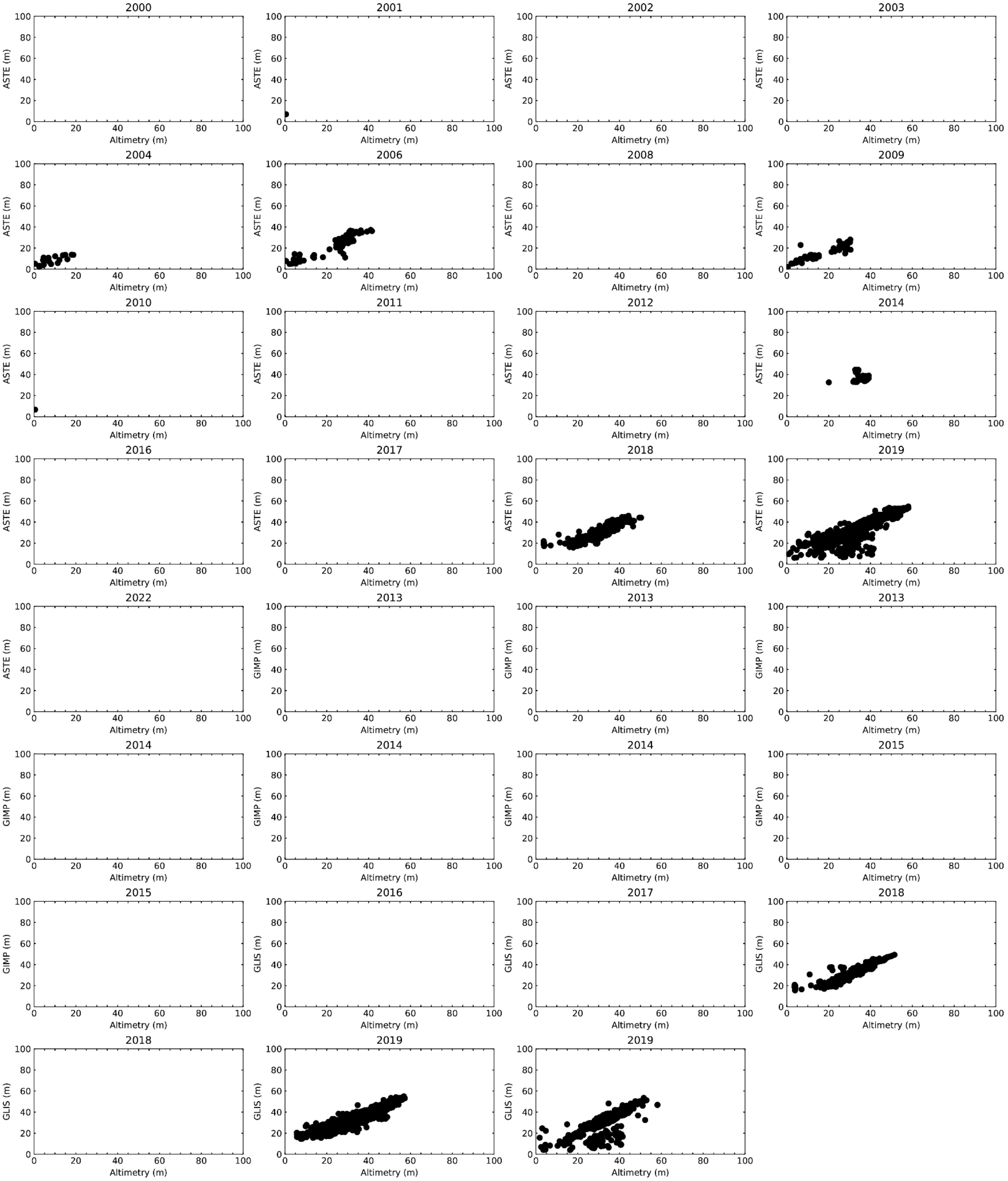

Fig. S38. Fit in surface elevation between ASTER (ASTE), GIMP and GLISTIN-A (GLIS) DEMs and yearly reference DEMs assembled using a combination of Altimetry data (see Material and Methods) over the Størestrommen ice shelf. Empty plot corresponds to years without overlap with altimetry data.

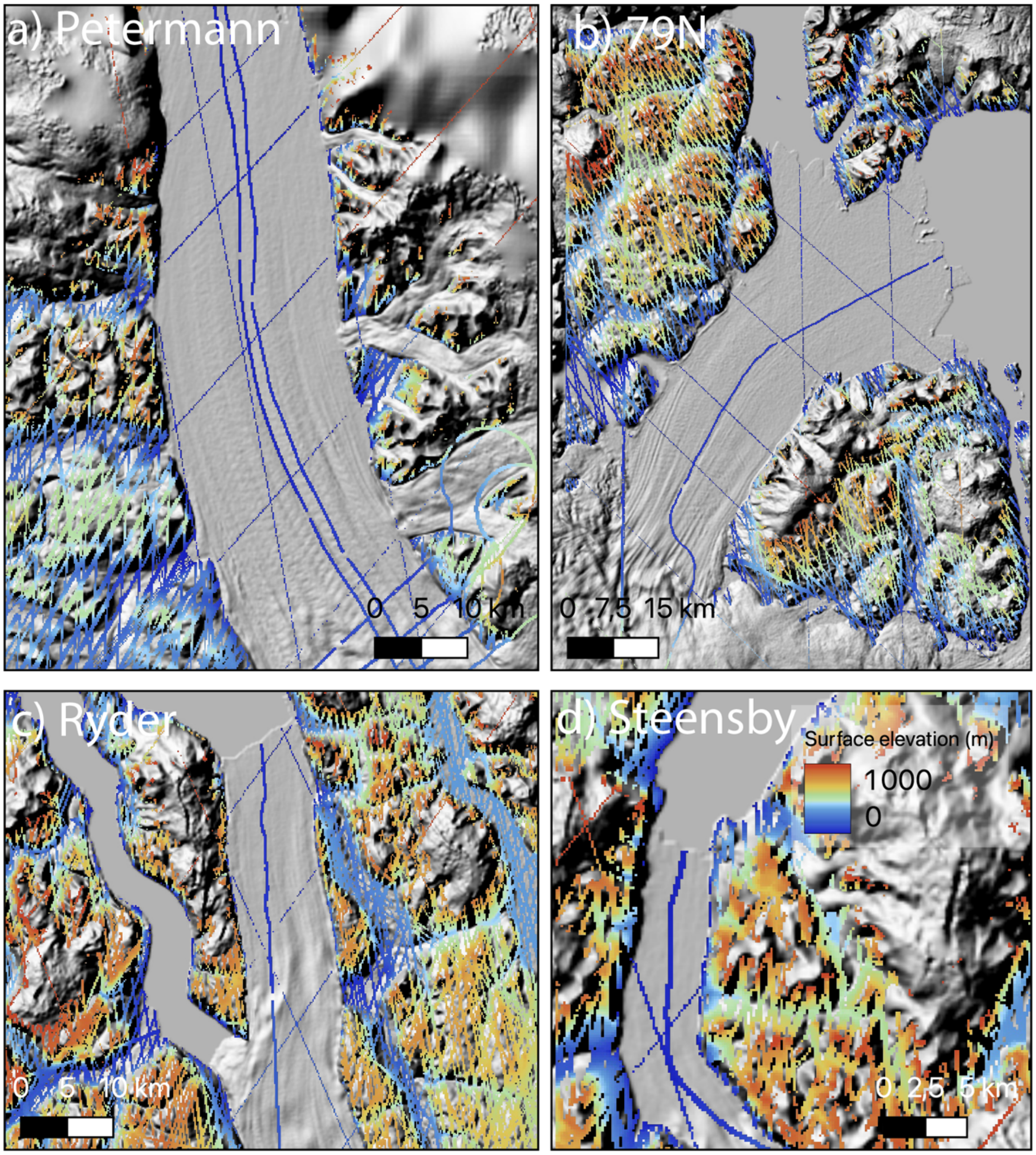

Fig. S39. Examples of reference DEMs used for co-registration in the year of 2009 for a) Petermann, b) 79N, c) Ryder and d) Steensby glaciers. Surface elevation is color coded from blue to red and overlaid on a shaded version of the digital elevation model from Bedmachine v3[2].

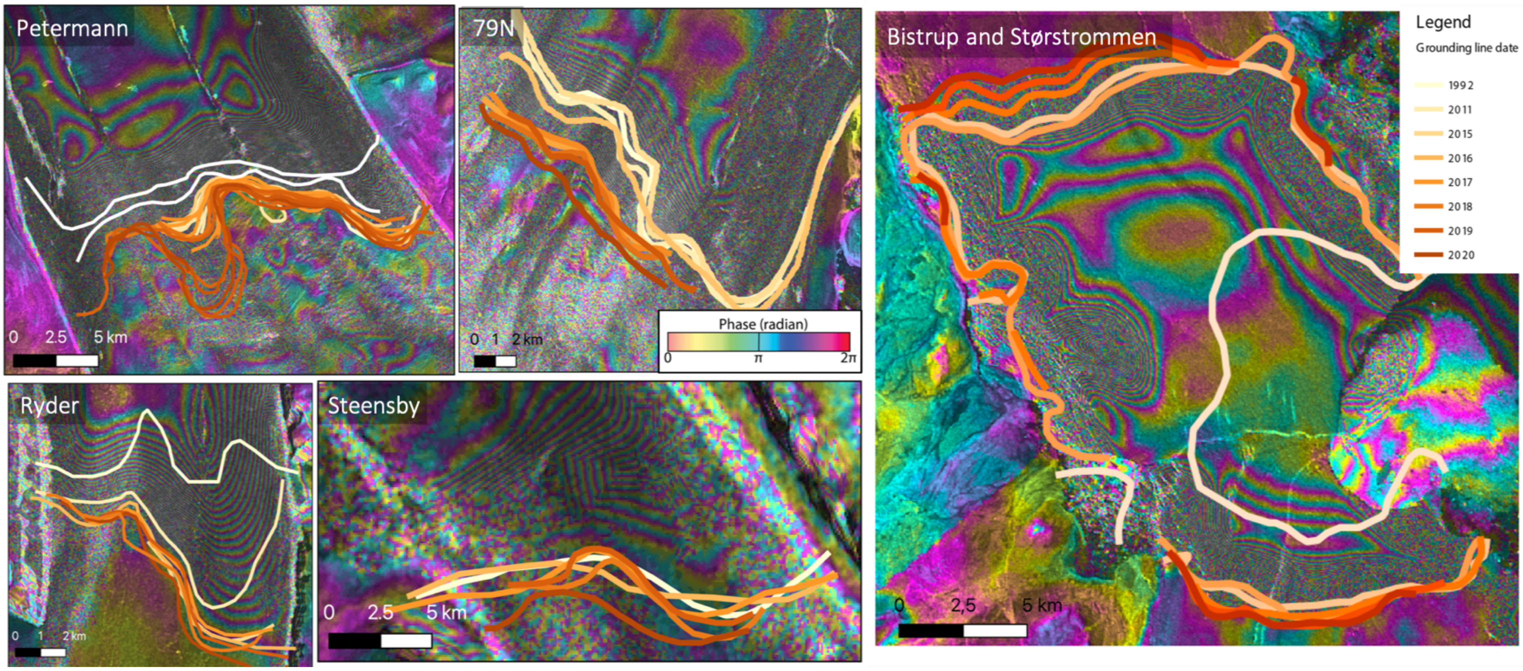

Fig. S40. Grounding line retreat for the studied ice shelves overlaid on double differential interferograms from year 2020.

Supplementary References:

1. Mouginot, J. *et al.* Forty-six years of Greenland Ice Sheet mass balance from 1972 to 2018. *Proc Natl Acad Sci USA* 201904242 (2019) doi:10.1073/pnas.1904242116.

2. Morlighem, M. *et al.* BedMachine v3: Complete Bed Topography and Ocean Bathymetry Mapping of Greenland From Multibeam Echo Sounding Combined With Mass Conservation. *Geophysical Research Letters* **44**, 11,051-11,061 (2017).
