# Supplementary material for: Rapid disintegration and weakening of ice shelves in North Greenland
Source: Nat Commun. 2023 Nov 7;14:6914. doi: 10.1038/s41467-023-42198-2 (PMC10630314; doi:10.1038/s41467-023-42198-2)
Supplement: Supplementary file 3 — Description of Additional Supplementary Files [file 41467_2023_42198_MOESM3_ESM.pdf]

Description of Additional Supplementary Files

File Name: Supplementary Data 1
Description: Time coverage of every datasets analyzed within this study. Columns A-B: Datasets names and specifications. Columns C to AU : years of coverage
